# Biodegradable Polyglycerols Combining Antioxidant Activity and Sulfation-Induced Complement Inhibition

**DOI:** 10.1021/acs.biomac.5c01615

**Published:** 2025-12-11

**Authors:** Hanna Koeppe, Daniel Horn, Jens Dernedde, Rainer Haag

**Affiliations:** † Institute of Chemistry and Biochemistry, 9166 Freie Universität Berlin, 14195 Berlin, Germany; ‡ Institute for Laboratory Medicine, Clinical Chemistry and Pathobiochemistry, 14903 Charité-Universitätsmedizin Berlin, 13353 Berlin, Germany

## Abstract

Polyglycerol platforms are promising for polymer therapeutics due to their multifunctionality and biocompatibility. Our aim was to introduce biodegradability as well as antioxidant properties to the polyglycerol backbone using cyclic comonomers with thioether and ester functionalities. Anionic ring-opening copolymerization of glycidol and either 1,4-oxathiepan-7-one or thiodiglycolic anhydride yielded the hyperbranched structures: GOTO or GTA, respectively. Characterization confirmed molecular weights of 10 kDa and the successful incorporation of 10 mol % comonomer while maintaining water solubility. Sulfated derivatives, GOTO-S and GTA-S, were obtained with a high degree of sulfation. All copolymers showed good cytocompatibility as well as degradability under physiological conditions. Significant antioxidant activity attributed to the thioether groups of the copolymers was demonstrated via the ABTS radical scavenging assay. GTA emerged as the strongest radical scavenger among the polymers tested, likewise, GTA-S outperformed GOTO-S. Notably, the sulfated derivatives effectively inhibit complement activation with potencies comparable to dPGS and heparin, demonstrating their potential for applications in oxidative stress-related inflammation.

## Graphical abstract

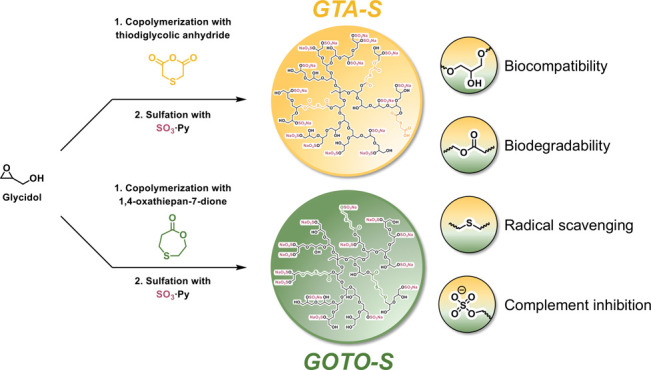

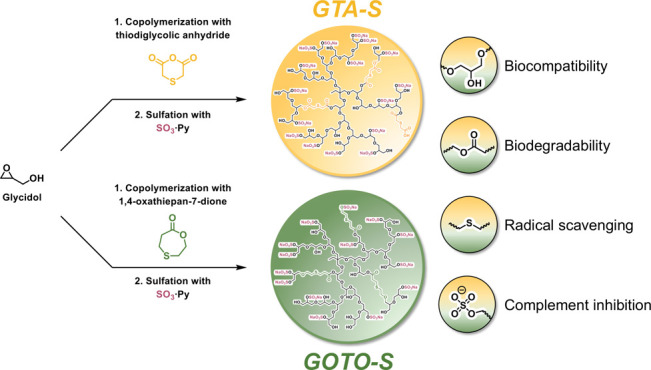

## 1 Introduction

Synthetic polymers are increasingly used in clinical medicine, with a growing number of newly approved polymer therapeutics.^1^ The majority of these contain poly­(ethylene glycol) (PEG), owing to its tried-and-tested safety profile and pharmacokinetic benefits.^2^ However, an increasing prevalence of drug-induced anti-PEG antibodies, as well as pre-existing immunity in treatment-naïve individuals,^3^ can lead to hypersensitivity reactions and accelerated blood clearance (ABC).^4^ Although the mechanisms are not yet fully understood, it is assumed to involve the formation of nanoparticle–antibody immune complexes that trigger complement activation and phagocytosis.^4^ This has led to concerns about safety (hypersensitivity) as well as efficacy (ABC) of PEG-containing formulations. At the same time, persistent reliance on PEG in both approved therapeutics and consumer products,^5^ alongside the growing public need for mRNA vaccines, has intensified the demand for the development of alternative stealth polymers.^6^

During the last decades, many potential PEG alternatives have been investigated, such as poly­(2-oxazoline), poly­(zwitterions), polysarcosines, and the well-established class of polyglycerols.^7–10^ Various architectures of the latter have been extensively explored for biomedical applications.^11,12^ Overall, polyglycerols combine excellent biocompatibility^13^ and low immunogenicity^14^ with a straightforward synthesis and multifunctionality.^15^ Several studies show that polyglycerol is a promising stealth polymer as it extends circulation half-life^16,17^ and does not induce ABC.^18,19^

However, similar to PEG, polyglycerol suffers from limited biodegradability, which can lead to bioaccumulation.^20,21^ In addition, polymers such as PEG and polyglycerol lack intrinsic therapeutic properties and do not respond to specific stimuli, limiting their application in complex disease environments. To address these shortcomings, our group and others have developed polyglycerol-based copolymers with hydrolytically labile ester functionalities (e.g., citric acid,^22^ lactide,^23^ succinic anhydride,^24^ caprolactone^25^), as well as reduction-sensitive disulfides,^26,27^ and a combination of both.^28^ These cleavable linkers enable site-specific degradation in response to environmental cues such as acidic pH, enzymatic activity, or elevated glutathione levels, conditions typically found in the tumor microenvironment.

For applications at inflammatory sites characterized by oxidative stress, the introduction of oxidation-sensitive moieties is highly desirable. A prominent example are thioethers, which have been widely employed as oxidation-responsive switches in drug delivery systems (DDS).^29,30^ Micellar DDS for example have been achieved through the design of block copolymers incorporating sulfur-rich blocks such as polythioethers.^31^ The concept is based on the oxidative transformation of thioethers into more hydrophilic sulfoxides or sulfones, which can trigger disassembly, swelling, and drug release. But structural incorporation of thioethers can also impart intrinsic antioxidant activity, as demonstrated by d’Arcy and co-workers.^32^ The linear polythioether poly­(thioglycidyl glycerol) (PTGG) provides protection of the therapeutic cargo and adjacent tissues by scavenging reactive oxygen species (ROS), thereby preventing oxidative damage. This behavior, termed “active stealth”, also reduces immune activation and extends circulation time, positioning PTGG as a functional alternative to conventional PEGylation.

Although chronic or excessive levels of ROS can cause tissue damage, ROS are involved in physiological signaling and host defense.^33,34^ So, the goal of therapeutic intervention is not to eliminate ROS, but to modulate redox homeostasis in a context-dependent manner. Despite the strong radical scavenging ability of many small molecule antioxidants, limitations like low stability, limited bioavailability, and short circulation times hinder efficient and continuous antioxidative effects.^35^ Functionalized polymeric antioxidants offer the potential of localized, gradual, and sustained ROS modulation making them promising for inflammatory disease settings.^36^

To mitigate inflammation, various polymeric strategies have been explored besides ROS-scavengers. Most commonly, as DDS to improve treatment efficacy while minimizing systemic side effects of antiinflammatory therapeutics, like corticosteroids or biotherapeutics.^37^ However, beyond their role as passive carriers, certain polymers have been shown to exert intrinsic antiinflammatory activity.^38^ A notable example here is dendritic polyglycerol sulfate (dPGS), originally introduced as a synthetic heparin mimetic with reduced anticoagulation activity but enhanced anticomplementary effects compared to its natural counterpart.^39^ Further studies revealed its multivalent binding to endothelial P-selectin and leukocytic L-selectin, thereby effectively reducing leukocyte extravasation. In vitro experiments demonstrated its ability to inhibit complement activation through binding of complement factors C3 and C5, while in vivo studies confirmed reduced levels of the pro-inflammatory mediator C5a, resulting in impaired leukocyte chemotaxis.^29,40^ Structure–activity relationships have also been explored, and dPGS has demonstrated therapeutic potential in various disease models, including neurological disorders,^41^ cancer,^42^ and arthritis.^43^ These findings underscore its promise as a multivalent, polymeric antiinflammatory agent.^44^

Based on these insights, we aimed to develop a multifunctional, degradable polyglycerol-based polymer that combines intrinsic antioxidant and antiinflammatory properties within a single scaffold. To realize this concept, we copolymerized glycidol with thioether-containing monomers via ring-opening polymerization, using either 1,4-oxathiepan-7-one (OTO), a ε-caprolactone derivative, or thiodiglycolic anhydride (TA). The resulting copolymers, GOTO and GTA, incorporate ester and thioether groups, which are designed to enable biodegradability and provide intrinsic antioxidative capacity. These effects are complemented by anti-inflammatory properties of the subsequently introduced sulfate groups. The resulting polymer systems thus merge stimuli-responsiveness with intrinsic bioactivity and hold potential as “active stealth” polymers for targeted drug delivery in inflammatory environments.

## 2 Materials and Methods

### 2.1 Materials

Acetonitrile, diethyl ether, cyclohexane, ethyl acetate (HPLC grade), hydrogen peroxide, sodium hydrogen carbonate, potassium hydroxide, and sodium chloride were purchased from Fisher Scientific. 1,1,1-Tris­(hydroxymethyl)­propane, glycidol, strontium isopropoxide, and potassium persulfate were purchased from Sigma-Aldrich. 4-Nitrophenol, acryloyl chloride, and thiodiglycolic anhydride were purchased from abcr. Potassium carbonate was purchased from Carl Roth. Triethylamine and dry methanol were purchased from Acros Organics. Pyridine-sulfur trioxide complex was bought from TCI. Dichloromethane and dry dimethylformamide were purchased from VWR Chemicals and Thermo Scientific, respectively. 2,2′-Azino-bis­(3-ethylbenzothiazoline-6-sulfonic acid) diammonium salt (ABTS) was purchased from Alfa Aesar. Ion-exchanged water was used unless stated otherwise. Milli-Q water was prepared by a purification system (Milli-Q Reference A+). Regenerated cellulose (RC) syringe filters were purchased from VWR international. Hyperbranched polyglycerol (*M*_n_ = 9.4 kDa; PDI = 1.3) was provided by Cathleen Schlesener. Dialysis tubings (MWCO = 1000 g mol^–1^) were purchased from Thermo Fisher Scientific. Flash column chromatography was carried out on a CombiFlash Rf+ from Teledyne Isco (Lincoln, Nebraska, USA). RediSep Silver normal phase disposable columns for flash chromatography (40 g Silica) were used. CCK-8 was purchased from Hycultec (Beutelsbach, Germany) and Wieslab Complement System MBL Pathway kit from Svar Life Science AB (Malmö, Sweden).

### 2.2 Analytical Methods

^1^H- and ^13^C NMR spectra were recorded on a Bruker AVANCE III (500 or 700 MHz, and 176 MHz) at 25 °C. Polymers were dissolved in D_2_O, monomers in DMSO-*d*_6_ and CDCl_3_. Chemical shifts δ were reported in ppm and referenced using the solvent peak as internal standard (δ = 4.79 ppm (D_2_O); 2.50 ppm (DMSO-*d*_6_): 7.26 ppm­(CDCl_3_). Gel permeation chromatography in DMF (HPLC-grade, Sigma-Aldrich; 10 mM of LiBr) was carried out on a customized system (PSS Polymer Standard Service GmbH, Mainz, Germany) coupled with a differential refractive index detector (PSS SECcurity RI), and 2 columns (PSS SDV linear M 5 μm; 5 and 30 cm). Polymer samples were dissolved in GPC eluent and swelled for 24 h before filtration using RC 0.45 μm syringe filters. Polymer solutions of 1.5 mg mL^–1^ with volumes of 50 μL were analyzed. Measurements were taken at 25 °C at flow rates of 1.0 mL min^–1^. PS standards were used for calibration. Infrared spectroscopy was carried out on a Bruker Alpha II FT-IR spectrometer with a resolution of 2 cm^–1^. The attenuated total reflection (ATR) technique was used in combination with a Ga/diamond crystal. Elemental analysis was carried out on an Elementar Vario EL. Samples between 0.8 and 3.0 mg were analyzed. Dynamic light scattering (DLS) and zeta potential was measured on a zetasizer (Nano ZS, Malvern Instruments Ltd., Worcestershire, U.K.) equipped with a HeNe laser (λ = 633 nm, NIBS, 4 mW). Polymer solutions with 2 mg mL^–1^ were prepared in PBS (150 mM, pH 7.4) for DLS or PB (10 mM, pH 7.4) for zeta potential. Solutions were filtered (RC 0.45 μm) prior measurements. Detection was carried out with a 173° scattering angle (backscatter). Differential scanning calorimetry was carried out on a differential scanning calorimeter (Netzsch, STA 449F3). Samples were added to an open Al_2_O_3_, 85 μL crucible. Temperature ranges between −80 and 120 °C were cycled 3 times at rates of 5.0 K min^–1^. Data of all cycles was numerically differentiated. Determination of the glass transition temperature was carried out using data from the second cycle. UV/vis absorption spectra were recorded on a LAMBDA 365 (PerkinElmer, Waltham, USA).

### 2.3 Synthesis of 1,4-Oxathiepan-7-one (OTO) Monomer

OTO was prepared following the procedure of Li et al. with modifications.^45^ Two reactions were performed sequentially (Scheme S1): First, 1.7 equiv KOH (12.9 g) was dissolved in water (6 mL mmol^–1^), followed by the addition of 1.0 equiv of *para*-nitrophenol (18.9 g). After cooling to 5 °C, 0.9 equiv acryloyl chloride (10.0 mL) was added dropwise, maintaining the temperature below 5 °C. The mixture was then stirred for 4 h. The crude product was obtained as a yellow precipitate. Purification was carried out by filtration with ice-cold water to yield a white solid (9.73 g, 41%). ^1^H NMR (500 MHz, DMSO): δ = 8.32 (m, 2H), 7.52 (m, 2H), 6.60 (dd, 1H), 6.45 (dd, 1H), 6.23 (dd, 1H) ppm.

The formed *para*-nitrophenyl acrylate was used in a one-pot, two-step reaction. 0.8 equiv mercaptoethanol (2.41 mL) were mixed with acetonitrile (17.6 mL, 0.5 mL per mL mercaptoethanol). Separately, 1.0 equiv of *para*-nitrophenol acrylate (6.66 g), 17 equiv triethylamine (4.82 mL), and 2.0 equiv K_2_CO_3_ (9.47 g) were dissolved in acetonitrile (343 mL, 10 mL mmol^–1^). The mercaptoethanol solution was then added dropwise over 12 h, and the mixture was stirred for additional 8 h at room temperature. Excess potassium carbonate was filtered off, and acetonitrile was removed under reduced pressure. The solid residue was dissolved in dichloromethane and filtered. The filtrate was concentrated under reduced pressure. The crude product was purified by flash column chromatography (30% v/v ethyl acetate in cyclohexane) and recrystallized twice in diethyl ether, yielding colorless crystals (2.50 g, 54%). ^1^H NMR (500 MHz, CDCl_3_): δ = 4.55 (m, 2H, C*H*_2_OC = O), 3.11 (m, 2H, C*H*_*2*_CH_2_C = O), 2.91 (m, 2H, C*H*_*2*_CH_2_OC = O), 2.79 (m, 2H, C*H*_*2*_C = O) ppm. HRMS (ESI): *m*/*z* calcd for C_5_H_8_NaO_2_S^+^ 155.0138, found 155.0144 [M + Na]^+^; *m*/*z* calcd for C_10_H_16_NaO_4_S_2_^+^ 287.0383; found, 287.0388 [2 M + Na] ^+^.

### 2.4 Synthesis of Hyperbranched GOTO and GTA Copolymers

The procedure was carried out as described bySunder et al.^46^ 1.0 equiv of 1,1,1-trimethylolpropane (TMP; 14.9 mg) was added to a Schlenk flask, melted at 65 °C and dried for 1 h in vacuo. Afterward, 0.4 equiv of strontium isopropoxide (9.1 mg) were added, the mixture was left to deprotonate for 30 min and then dried at reduced pressure for 1 h. The mixture was heated to 100 °C before adding glycidol (1.0 mL) and 1,4-oxathiepan-7-one (22.1 mg) in a molar ratio of 9:1. Reactions were stirred at 100 rpm for 3 days, and terminated by quenching with DMF (5 mL) and water (20 mL). Crude products were dialyzed against water for 3 days. The final product GOTO was obtained after freeze-drying as slightly yellowish honey-like mass (530.6 mg, 40%). ^1^H NMR (700 MHz, D_2_O): δ = 4.50–3.50 (mm, 5H, G; 2H, OTO), 3.00–2.50 (mm, 6H, OTO), 1.60–1.20 (m, 2H, TMP), 1.00–0.70 (m, 3H, TMP). GPC (DMF): *M*_n_ = 11.7 kDa, *M*_w_ = 16.6 kDa, *D̵* = 1.4. EA (%): C 48.37, H 8.147, N 0.034, S 4.059.

GTA was synthesized following the same procedure with thiodiglycolic anhydride (TA, 22.1 mg) and isolated as orange honey-like viscous mass (479.5 mg, 36%). ^1^H NMR (700 MHz, D_2_O): δ = 4.50–3.50 (mm, 5H, G), 3.65–3.55 (mm, 4H, TA), 1.60–1.20 (m, 2H, TMP), 1.00–0.70 (m, 3H, TMP). GPC (DMF): *M*_n_ = 10.2 kDa, *M*_w_ = 13.1 kDa, *D̵* = 1.3. EA (%): C 48.67, H 8.671, N 0.025, S 4.299.

### 2.5 Determination of *M*_n_ by ^1^H NMR End-Group Analysis

The number-average molecular weight (*M*_n_) of the synthesized hyperbranched copolymers was determined by means of ^1^H NMR spectroscopy using end-group analysis. This method uses the integral of an initiator signal as reference to calculate the number of repeating units (RU) of the monomers based on the integral intensities of their signals. The methyl group of the TMP initiator served as reference, since every molecule of GOTO or GTA possesses exactly one unit of TMP. Accordingly, the integral of the TMP signal at 1.0–0.8 ppm was set to 3 for the calculation of *M*_n_. In the case of GOTO, the integrated signal intensity of six methylene protons of OTO at 3.0–2.5 ppm was divided by six to give the RUs of OTO per polymer molecule. The signal of the remaining two OTO methylene protons overlaps with the polyglycerol backbone signal at 4.4–3.5 ppm. To obtain the RUs of G (5H), this mixed integral was corrected by subtracting the calculated integral contribution of OTO and subsequently divided by five. For GTA, the signal of all four TA methylene protons at 3.6 ppm overlaps with the polyglycerol backbone at 4.4–3.5 ppm preventing determination of the RUs of TA. Therefore, the TA content of 10.8 mol % determined by EA was used to calculate the contribution of TA and G to the intersecting integral. The number of RUs of each monomer was multiplied by their molecular weight to obtain the *M*_n_ of the copolymer.

### 2.6 Sulfation of GOTO and GTA Copolymers

Previously synthesized polyglycerol-based copolymers were functionalized following the method described by Haag et al.^39^ 1.0 equiv of dried GOTO (207.0 mg) copolymer was dissolved in dry DMF (5 mL). In another Schlenk flask, a solution of 2.0 equiv sulfur-trioxide-pyridine complex (737.5 mg) in dry DMF (2 mL mmol^–1^) was prepared and added dropwise to the polymer solution while stirring at 70 °C. After 24 h, the reaction was quenched with ice-cold water, and the pH was adjusted to 7 using NaHCO_3_ (2 M). The solution was transferred to a dialysis bag and dialyzed against brine for 24 h. The sodium chloride concentration was then gradually reduced before dialyzing against distilled water for another 24 h. Lyophilization yielded the sulfated copolymer GOTO-S as fluffy white, solid (391.3 mg, ≥98%). ^1^H NMR (700 MHz, D_2_O): δ = 4.75–3.30 (mm, 5H, G; 2H, OTO), 3.25–2.30 (mm, 6H, OTO), 1.65–1.25 (m, 2H, TMP), 1.02–0.86 (m, 3H, TMP). EA (%): C 21.78, H 3.52, N 0.23, S 15.42.

GTA-S was synthesized following the same procedure using GTA (185.0 mg) and sulfur-trioxide-pyridine complex (665.9 mg) and subsequently isolated as fluffy slightly yellowish solid (348.4 mg, 97%). ^1^H NMR (700 MHz, D_2_O): δ = 4.75–3.35 (mm, 5H, G; 4H, TA), 1.65–1.25 (m, 2H, TMP), 1.05–0.80 (m, 3H, TMP). EA (%): C 21.10, H 3.05, N 0.13, S 15.55.

### 2.7 Degradation Study

Degradation experiments of GTA and GOTO were performed in duplicates under physiologically relevant conditions (37 °C, DPBS at pH 7.4) by incubating copolymer (120 mg) in DPBS (28 mL) in Falcon tubes in a shaking incubator for 4 weeks. Aliquots (3.5 mL) were taken and lyophilized at time points 0, 1, 2, 3 days, and after 1, 2, 3, and 4 weeks. The freeze-dried samples were dissolved in deuterium oxide and ^1^H NMR spectra (500 MHz) were recorded. For quantitative analysis the TMP signal at 1.02–0.78 ppm was normalized to 3 (internal reference) and the integral of the carboxylic acid methylene signal at 2.60–2.38 ppm (GOTO) and 3.52–3.26 ppm (GTA) was monitored over time. Ester cleavage was calculated from the increase in the carboxylic acid integral (relative to 0 d time point) using eq S3.

### 2.8 Cell Viability Assay

All cell experiments were conducted according to German genetic engineering laws and German biosafety guidelines in the laboratory (safety level 2). Cell viability was determined using a Cell Counting Kit-8 (CCK-8) according to the manufacturer’s instructions. HeLa and RAW 264.7 cells were obtained from the Leibniz-Institut DSMZ—Deutsche Sammlung von Mikroorganismen und Zellkulturen GmbH and cultured in DMEM supplemented with 10% (v/v) FBS, 100 U mL^–1^ penicillin and 100 μg mL^–1^ streptomycin. Cells were seeded in a 96-well plate at a density of 5 × 10^4^ cells mL^–1^ in 90 μL DMEM per well overnight at 37 °C and 5% CO_2_. Ten μL of sample (dissolved in Milli-Q water) were added in serial dilutions including positive (1% SDS) and negative controls (DMEM, Milli-Q water) and incubated for another 24 h at 37 °C and 5% CO_2_. For background subtraction, wells without cells and samples in medium were used. After 24 h of incubation, the CCK-8 solution was added (10 μL per well), and the absorbance (450 nm/650 nm) was measured after approximately 3 h of incubation of the dye using a Tecan plate reader (SPARK, Tecan Group Ltd.). Measurements were performed in triplicate, and the experiments were repeated three times. Cell viability was calculated by setting the negative control to 100% and the cell-free control to 0% after subtracting the background signal.

### 2.9 Ex Vivo Red Blood Cell (RBC) Hemolysis Assay

Concentrated human erythrocytes for research purposes (serum separated) were purchased from German Red Cross (DRK-Blutspendedienst Nord-Ost). Hemocompatibility of the copolymers was evaluated according to a published protocol.^47^ Briefly, red blood cells (RBCs) were washed, and a 1:25 RBCs suspension (190 μL) was coincubated at 37 °C for 1, 4, and 24 h with different concentrations of GOTO, GTA, GOTO-S, and GTA-S (10 μL). DPBS (10 μL) and 1% Triton X-100 (10 μL) served as negative and positive controls, respectively. To correct for polymer self-absorbance, reference samples containing the same polymer concentrations in DPBS without RBCs were measured in parallel and subtracted from the corresponding samples. After incubation, the plate was centrifuged, and 100 μL of supernatant was transferred to a flat 96-well plate for absorbance measurement at 540 nm (Tecan SPARK, Tecan Group Ltd.). Hemolysis (%) was calculated after background correction (DPBS blank) and normalization to the positive control. All measurements were performed in triplicate.

### 2.10 ABTS Radical Scavenging Assay

The principle of the assay is based on the absorbance of the generated stable ABTS radical cation (ABTS^•+^). The blue-green ABTS^•+^ can be reduced by antioxidants to the colorless ABTS. This decolorization process can be monitored via UV/vis spectroscopy and is directly proportional to the radical scavenging capacity.

The assay was performed according to a published procedure with minor modifications.^48^ Solutions of ABTS (7 mM) and potassium persulfate (20 mM) in water were mixed, to give final concentrations of 5 mM ABTS and 2.5 mM K_2_S_2_O_8_. After incubation in the dark for 12 h the blue-green ABTS radical cation (ABTS^•+^) was generated. Different ABTS^•+^ concentrations in 50 mM DPBS were screened to determine a concentration with absorbance values of 0.8–1 at 415 nm. DPBS (150 mM) and Milli-Q water were used for dilution. For this experiment, an ABTS^•+^ concentration of 50 μM was selected.

Aqueous stock solutions of all polymers were prepared with equimolar thioether concentrations (48 mM). dPG and dPGS were used in comparable concentrations (38.6 mg/mL, 76.4 mg/mL). The polymers were applied at a 1:100 dilution in the assay mixture, resulting in a final thioether concentration of 0.48 mM. Vitamin C (50 μM) served as positive control and an ABTS^•+^ solution (50 μM) as negative control. In detail, water (970 μL) and sample stock solution (30 μL) were mixed with DPBS (150 mM, 1 mL). Immediately before measurement, a stock solution of ABTS^•+^ (150 μM, 1 mL) in water was added to get a final assay volume of 3 mL in DPBS (50 mM). 90 s after addition, the measurement was started and the UV/vis absorption at 415 nm was monitored every 90 s. After 24 h, full UV/vis spectra from 200 to 700 nm were recorded for all samples as well as corresponding controls, and photos of the cuvettes were taken. The data was baseline corrected and the absorbance of the sample solutions without ABTS was subtracted from the corresponding sample measurements. Corrected absorbance values were plotted against time. The radical scavenging activity resulted from the percentage reduction in absorption relative to the negative control.

### 2.11 Complement Activation via MBL Pathway

The effect of the copolymers on complement system activity via the mannose-binding lectin pathway (MP) was evaluated using the enzyme-linked immunosorbent assay (ELISA)-based Wieslab Complement System MBL Pathway kit (Svar Life Science AB, Malmö, Sweden). Human serum was diluted 1:101 with MP diluent serving as positive control for complement activation (100%), while buffer controls (MP diluent) served as negative control (0%). Test compounds included nonsulfated polymers (GTA and GOTO), their corresponding sulfated analogs (GTA-S and GOTO-S), and control polymers previously reported (dPG and dPGS).^40^ Heparin (∼15 kDa, Calbiochem, Merck KGaA, Darmstadt, Germany) was used as reference control for the inhibition.^49^ All compounds were preincubated with the diluted human serum at various final concentrations (31.25, 62.5, 125, 250, 500, and 1000 nM) for 5 min at room temperature. After distributing 100 μL into each well, samples were incubated for 1 h at 37 °C. Subsequently, the supernatant was removed, wells were washed three times with washing buffer and then incubated with 100 μL of alkaline phosphatase-labeled antibodies for 30 min at room temperature to detect the immobilized membrane attack complex (MAC). Following three additional washing steps, 100 μL of substrate solution was added and incubated for 30 min at room temperature. The absorbance of each well was measured in a plate reader (Tecan, Infinite 200 PRO Microplate Reader, Männedorf, Switzerland) at 405 nm. Buffer controls (MP diluent) were subtracted from each measurement, and all test compound measurements were normalized to the untreated diluted human serum (positive control, 100%) to obtain complement system activity. The potency of inhibitors was expressed as IC_50_ value, which represents the inhibitor concentration required to reduce complement activity by 50%.

## 3 Results and Discussion

### 3.1 Synthesis and Characterization of Hyperbranched Polyglycerol Copolymers GTA and GOTO

Two distinct hyperbranched polyglycerol copolymers, GOTO and GTA, were synthesized by anionic ring-opening polymerization of glycidol (G) and a thioether-containing comonomer. GTA was obtained using the commercially available thiodiglycolic anhydride (TA). For the synthesis of GOTO, the comonomer 1,4-oxathiepan-7-one (OTO) was prepared. Optimization of the literature-known OTO synthesis through a lower reaction temperature and pseudodilution conditions successfully minimized the formation of undesired dimers and oligomers in the second step, resulting in improved monomer yield and purity (Scheme S1 and Figure S1).

GOTO and GTA copolymers with varying thioether content were synthesized by adjusting the monomer ratios of glycidol and the respective comonomer (Scheme 1). We prioritized maximized incorporation of thioether groups, as their number was considered the key factor dictating the radical-scavenging behavior of the copolymers. GOTO copolymers with 5, 10, and 20 mol % comonomer units were successfully synthesized. Due to insufficient water solubility, GOTO with 20 mol % comonomer was excluded. For GTA, the highest achievable incorporation of the thioether-containing comonomer was approximately 10 mol %. To assess the influence of the incorporated OTO and TA segments, copolymers with 10 mol % were selected for further comparative studies alongside hyperbranched polyglycerol (dPG).

**Scheme 1:**
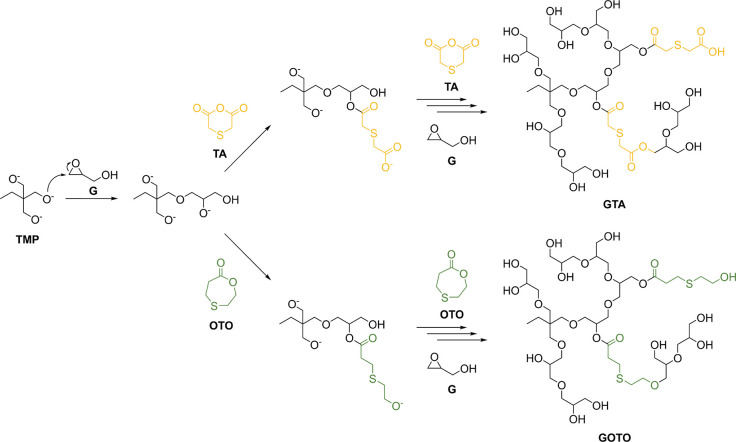
Polymerization of Glycidol (G) with Thiodiglycolic Anhydride (TA, Yellow) or 1,4-Oxathiepan-7-one (OTO, Green) Initiated by Deprotonated 1,1,1-Trismethylolpropane (TMP) to form GTA and GOTO Copolymers

The incorporation of OTO and TA comonomer units into the PG backbone was qualitatively confirmed through IR spectroscopy. For this purpose, the characteristic and strong carbonyl stretching vibration band of the ester groups contained in the comonomer segments was analyzed. IR spectra of GOTO and GTA were recorded and compared to dPG, which contains no ester bonds. In contrast, each OTO unit contains one ester group, while each TA unit consists of two ester groups. These structural differences are reflected in the intensity of the C=O band at approximately 1725 cm^–1^ in the respective IR spectra (Figure 1c). This qualitatively proves the successful incorporation of the comonomers and illustrates the different ester group density in GOTO and GTA.

**Figure 1:**
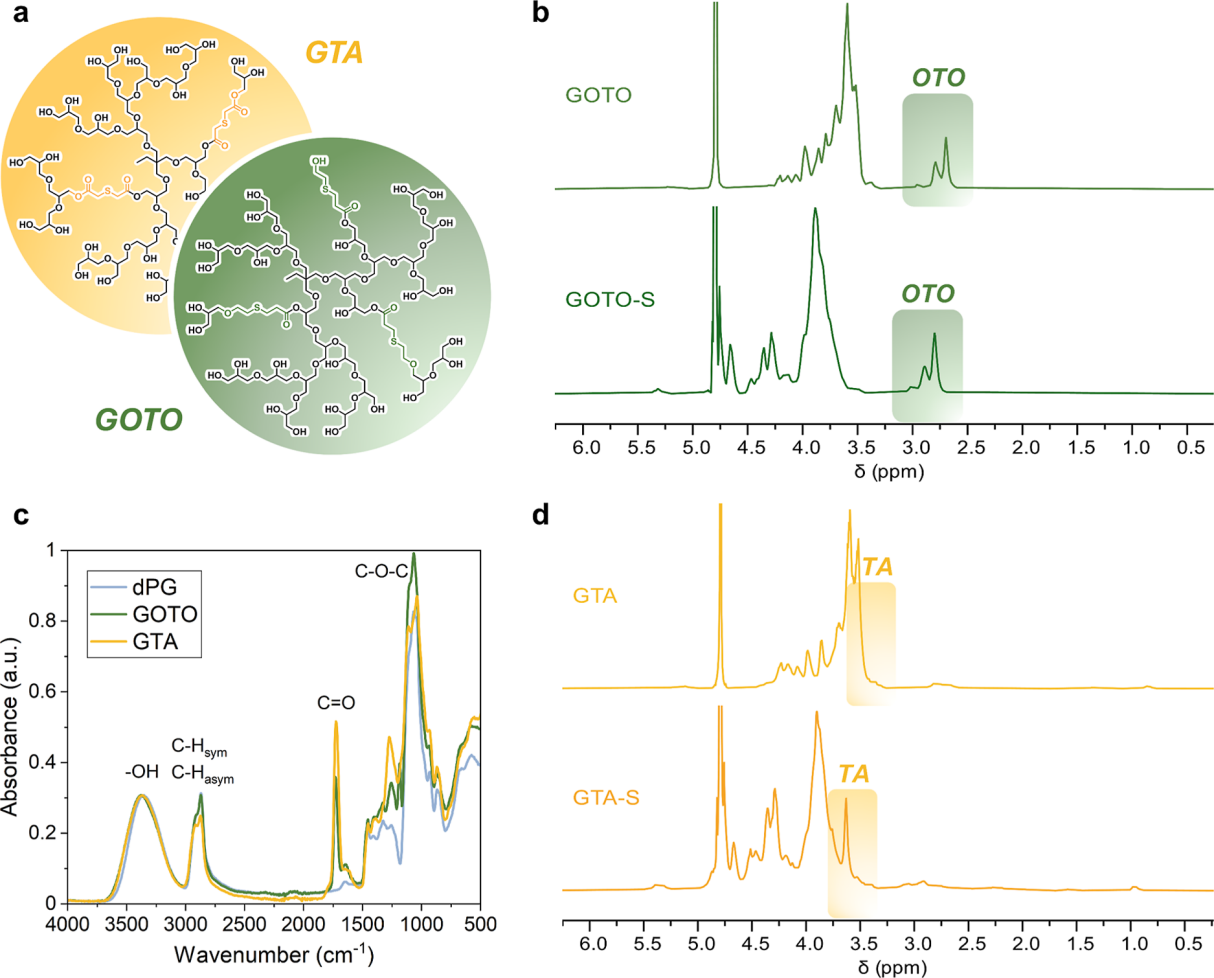
(a) Exemplified copolymer structure of GOTO and GTA. ^1^H NMR spectra of (b) GOTO and GOTO-S as well as (d) GTA and GTA-S in D_2_O. (c) IR spectra of dPG, GOTO, and GTA.

Two-dimensional NMR techniques, including Heteronuclear Multiple Bond Correlation (HMBC), were used to verify the covalent incorporation of the comonomers into the polyglycerol structure (Figure 2). The HMBC spectrum of GOTO reveals coupling between the carbonyl carbon of the OTO segments (174 ppm) and protons of the polyglycerol segments (4.3–4.1 ppm), as well as between methylene protons of OTO (2.9–2.7 ppm) and carbons of polyglycerol (71–65 ppm). For GTA, the coupling between the carbonyl carbons of TA (172 ppm) and protons of polyglycerol (4.3–4.2 ppm) could be clearly assigned. These results are supported by similar diffusion coefficients obtained from DOSY NMR experiments (Supporting Information) confirming the successful covalent binding between the comonomer units and the polyglycerol backbone.

**Figure 2:**
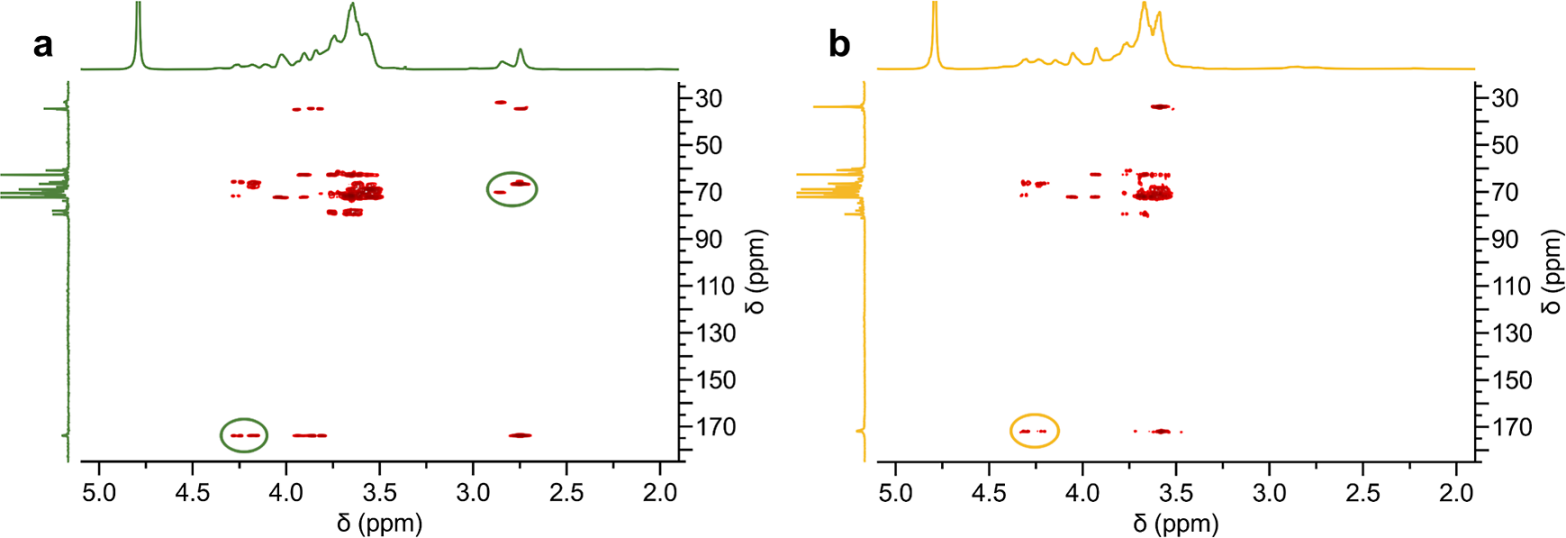
^1^H–^13^C-HMBC NMR spectrum of (a) GOTO and (b) GTA in D_2_O. The coupling signals between glycerol and comonomer units are encircled.

The ^1^H NMR spectrum of GOTO was used to quantify the incorporated OTO units (Figure 1b). Integrals corresponding to six OTO methylene protons (3.0–2.5 ppm) were used to determine the OTO content (9.7 mol %). For GTA, the signal of the methylene protons in the incorporated TA units (3.6 ppm) significantly overlaps with the polyglycerol backbone signals (4.4–3.5 ppm). This downfield shift is attributed to the deshielding effect of the adjacent carbonyl and thioether groups within the TA units. This signal overlap prevented accurate quantification by ^1^H NMR (Figure 1d). Therefore, elemental analysis (EA) was employed to determine the comonomer content for both copolymers, yielding 10.1 mol % for GOTO and 10.8 mol % for GTA (Table 1). These values were comparable to each other and to the aimed thioether content of 10 mol %.

**Table 1:** Characterization Overview of Synthesized Copolymers GOTO and GTA, as Well as Sulfated Derivatives GOTO-S and GTA-S.^a^ ^,^ ^b^

characterization parameter	GOTO	GOTO-S	GTA	GTA-S
comonomer content (mol %) by ^1^H NMR	9.7			
comonomer content (mol %) by EA	10.1		10.8	
*M*_n_ (kDa) by ^1^H NMR	10.6	24.9^d^	9.9	18.6^d^
*M*_n_ (kDa) by GPC	11.7	22.6^d^	10.2	22.4^d^
*D̵* by GPC	1.4		1.3	
hydrodynamic diameter (nm) by DLS	4.1 ± 0.2	4.0 ± 0.4	3.8 ± 0.2	3.9 ± 0.4
zeta potential (mV)	–8.6 ± 2.0	–24.7 ± 0.9	–16.1 ± 1.7	–24.8 ± 0.8
degree of branching^b^	0.42		0.4	
degree of sulfation^c^ (%)		80		81

a *D̵* = dispersity.

b Degree of branching (DB) was calculated from an inverse-gated ^13^C NMR analysis.

c Degree of sulfation (dS) was determined from elemental analysis (EA).

d *M*_n_ calculated based on the *M*_n_ of the polymer and the dS.

Number-average molecular weights (*M*_n_) of 10.6 kDa for GOTO and 9.9 kDa for GTA were determined by ^1^H NMR end-group analysis and confirmed the initially aimed molecular weights. Furthermore, gel permeation chromatography (GPC) was performed in DMF to determine the molecular weight distribution. The GPC results are with a *M*_n_ of 11.7 kDa for GOTO and 10.2 kDa for GTA in agreement with the molecular weights determined by ^1^H NMR in D_2_O. Polystyrene (PS) standards were used for GPC calibration, which may slightly overestimate absolute *M*_n_ values due to the chemical and structural difference between PS and branched PG-based polymers. Nevertheless, the agreement between GPC and ^1^H NMR results supports the reliability of the molecular weight data.

The degree of branching (DB) of GOTO and GTA was determined using inverse-gated ^13^C NMR spectroscopy (Figures S2 and S3). Hyperbranched polyglycerol (dPG) is a homopolymer consisting of AB_2_-type glycidol units, which were connected via a random polymerization mechanism. This results in linear, dendritic, or terminal units and a DB in the range of 0.53–0.59.^46^ In contrast to pure dPG, the GOTO and GTA copolymers incorporate additional linear units introduced by the comonomers. Consequently, their DB was expected to be lower than that of dPG. Following the method established by Hölter and co-workers, the calculation for GOTO and GTA via eq S2 yielded DB values in the range of 0.40–0.42.^50^ These values suggest the successful formation of hyperbranched structures, albeit with a lower degree of branching compared to dPG.

Molecular weight and DB influence the pharmacokinetic behavior of polymers. Higher molecular weight dPG has been shown to prolong circulation time, and dPG generally outperforms lPG and PEG of similar size.^16,51^ However, linear PG architectures exhibit superior virus inhibition, as multivalent inhibitors rely not only on binding affinity but also on steric shielding which is enhanced by polymer flexibility.^52–54^ Tully et al. compared PEG-, lPG, and dPG-protein conjugates, revealing that dPG is less effective in protein shielding, suggesting that increased flexibility improves shielding and may reduce immune clearance as a consequence.^17^ These findings illustrate the nuanced impact of molecular architecture on biological performance and provide context for the comparatively low DB observed in GOTO and GTA.

Differential scanning calorimetry (DSC) was performed to determine the glass transition temperature (*T*_g_) of the synthesized polymers, providing insight into their chain mobility. *T*_g_ was monitored through changes in heat flow while transitioning from a rigid glassy to a more flexible state (Figure S4). A comparative DSC analysis of dPG and GOTO copolymers with 10 mol % and 20 mol % OTO units revealed a systematic decrease in *T*_g_ with increasing OTO content. The *T*_g_ of dPG was observed at −56 °C, while GOTO with 10 mol % and 20 mol % OTO showed *T*_g_ values of −57 °C and −58 °C, respectively. This modest decrease in *T*_g_ suggests a subtle increase in polymer chain flexibility upon incorporation of the OTO units.

The colloidal stability of GOTO and GTA was investigated with dynamic light scattering (DLS) and zeta potential measurements. DLS measurements in DPBS at pH 7.4 showed hydrodynamic diameters of 4.1 nm for GOTO and 3.8 nm for GTA (Figure S5). These values confirm the unimolecular nature of the copolymers under these conditions. Zeta potential measurements in PB at pH 7.4 revealed differences in surface charge (Table 1). GTA exhibited a more negative surface potential (−16.1 mV) compared to GOTO (−8.6 mV) and dPG (−8.7 mV). This is attributed to the presence of carboxylic acid groups in the terminal TA segments of GTA. The comparable zeta potentials of GOTO and dPG reflect their similar surface functionality. Collectively, the DLS and zeta potential data demonstrates good colloidal stability of GOTO and GTA under physiological conditions.

### 3.2 Sulfation of GOTO and GTA Copolymers

Following the synthesis and characterization of GOTO and GTA, the copolymers were sulfated (Scheme S2) to introduce negative charge and associated biological functionalities, such as targeting and antiinflammatory effects, previously reported for dPGS.^29,39^ The degree of sulfation (DS) for the sulfated copolymers, GOTO-S and GTA-S, as well as dPGS was calculated from the elemental analysis (EA) with eq S1. Comparable high DS of 80% for GOTO-S, 81% for GTA-S, and 87% for dPGS were obtained (Table 1). DLS measurements of the copolymers showed no indication of aggregation or agglomeration with hydrodynamic diameters of 4.0 and 3.9 nm for GOTO-S and GTA-S. Zeta potential measurements proved stronger negative surface charges for all sulfated polymers compared to their nonsulfated analogs (Table 1). Collectively, these results confirm successful functionalization with sulfate groups and demonstrate the introduction of negative charges to the polymer surface. This represents a key characteristic associated with the biological functionality, previously described for dPGS.

### 3.3 Biocompatibility

To evaluate the potential of the nonsulfated and sulfated copolymers for therapeutic applications, cell viability of HeLa and RAW 264.7 cells was assessed using a CCK-8 assay. Both cell lines maintained high viability (>80%) after 24 h incubation with copolymer concentrations up to 1 mg mL^–1^ (Figure 3a,b), with no significant differences between sulfated and nonsulfated derivatives, indicating good cytocompatibility across all copolymers. To further assess hemocompatibility, an ex vivo red blood cell (RBC) hemolysis assay was conducted.^47^ This assay quantifies spectrophotometrically the release of hemoglobin into the supernatant as an indicator of membrane disruption. Hemolysis levels after 1 h of incubation with the copolymers (Figure 3c) were comparable to the negative control (DPBS) and remained below 2% after 4 and 24 h (Figure S8). Together, these findings demonstrate that the copolymers are nonhemolytic and well tolerated in vitro, indicating overall good biocompatibility.

**Figure 3:**
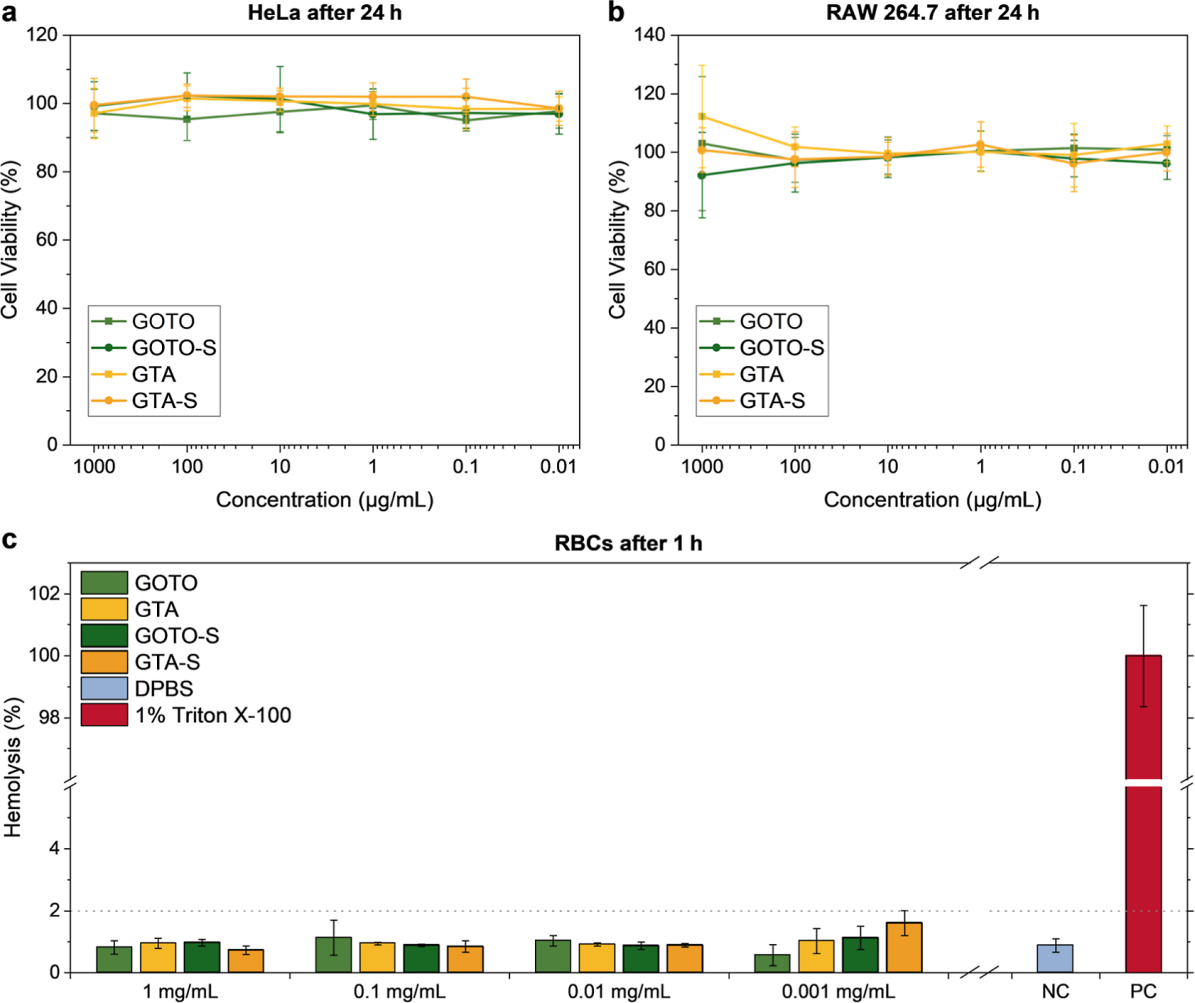
Cell viability of (a) HeLa cells and (b) RAW 264.7 cells after 24 h incubation with the copolymers (GOTO, GOTO-S, GTA, and GTA-S), determined by CCK-8 assay. (c) Ex vivo red blood cell (RBC) hemolysis after 1 h incubation with different copolymer concentrations, together with DPBS (negative control, NC) and 1% Triton X-100 (positive control, PC). Data are shown as mean ± SD, *n* = 3 (technical triplicates).

### 3.4 Degradation Study

The ester bonds introduced by the comonomers dictate the degradation behavior of the copolymers, a relevant property for in vivo applications. The hydrolytic degradation of GOTO and GTA was therefore investigated under physiological conditions (DPBS, 37 °C) for up to 4 weeks. ^1^H NMR spectroscopy was used to monitor changes over time (Figures 4a and S6). The increase of the signals at 2.50 ppm (GOTO) and 3.40 ppm (GTA) was attributed to the progressing hydrolysis and assigned to protons of the respective carboxylic acid degradation fragments (Figure 4b,c). The integrals of these signals were used to calculate the ester cleavage at the defined time points (eq S3). The resulting degradation profile of the copolymers followed a saturation curve (Figure 4d), reaching 44% degradation for GOTO and 63% for GTA after 4 weeks.

**Figure 4:**
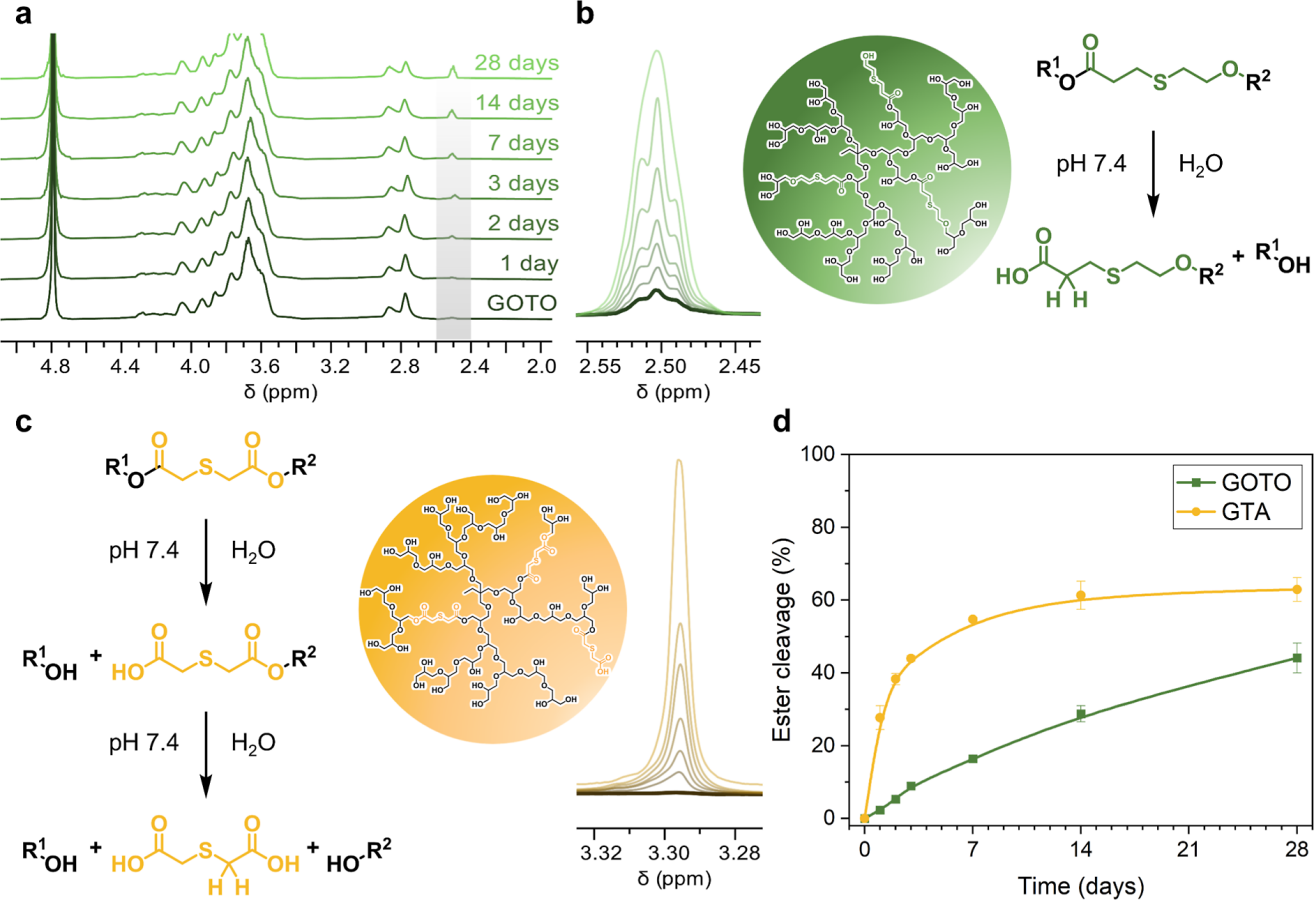
Degradation of GOTO and GTA in DPBS (pH 7.4) at 37 °C observed by ^1^H NMR spectroscopy after different time periods. (a) Stacked ^1^H NMR spectra of GOTO. Magnification of the signal associated with the degradation products of (b) GOTO and (c) GTA as well as schematic representations and hydrolysis mechanisms. (d) Time course of ester hydrolysis of GOTO and GTA, calculated from the increasing integral of the degradation products. Representative data from one of two independent experiments (Mean ± SD, *n* = 2).

The results demonstrate the hydrolytic susceptibility of the ester bonds within the copolymer backbones under the tested conditions. For the comparison between GOTO and GTA, the different number of ester groups is important. Since one TA unit contains two ester bonds, statistically, at least one ester bond per TA unit was cleaved during the study period. The observed hydrolytic degradation is a relevant property for biomedical applications, indicating potential biodegradability of the copolymers. While these in vitro results confirm inherent hydrolytic lability, in vivo degradation is anticipated to proceed at a faster rate, driven by enzymatic activity, acidic microenvironments, and oxidative processes involving the thioether groups.

OTO has previously been employed by Li and co-workers for the synthesis of its homopolymer and amphiphilic block copolymers. Their detailed investigation revealed that oxidation of the thioether accelerates ester hydrolysis and hence degradation compared to polycaprolactone (PCL) in physiological environments.^45^

### 3.5 ABTS Antioxidant Activity Assay

The antioxidant properties of GOTO, GTA, GOTO-S, and GTA-S were assessed using the ABTS radical scavenging assay. With this method, the antioxidant activity is determined by the ability of a compound to scavenge ABTS^•+^ radicals.^48^ All copolymers exhibited time-dependent radical scavenging activity, reaching complete scavenging comparable to the positive control Vitamin C (Figures 5c and S7), while dPG and dPGS exhibited no radical scavenging activity. This result was also visually observed, as solutions containing GOTO, GTA, GOTO-S, GTA-S, and Vitamin C became colorless within 24 h, in contrast to the blue solutions of dPG and dPGS, similar to the negative control water (Figure 5b). After 30 min, the nonsulfated copolymers GOTO and GTA reached 45% and 89% radical scavenging, respectively, whereas their sulfated counterparts GOTO-S and GTA-S showed substantially lower values of 22% and 50%. This reduced scavenging rate of the sulfated copolymers can likely be attributed to electrostatic repulsion between negatively charged sulfonate groups of the ABTS radical and the polysulfates.

**Figure 5:**
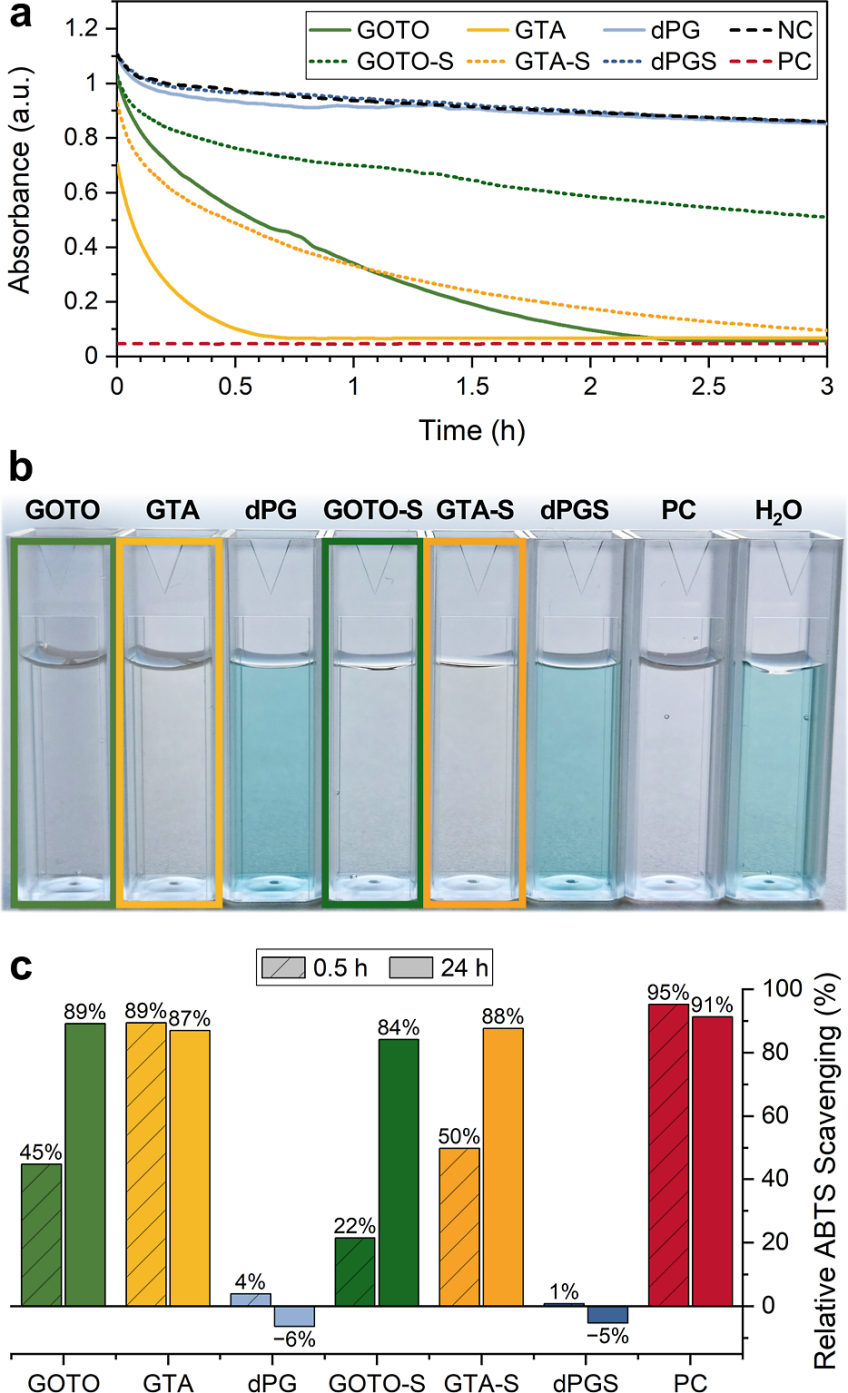
ABTS radical scavenging by the copolymers as well as dPG and dPGS, quantified by UV/vis absorption spectroscopy. Vitamin C (50 μM) served as a positive control (PC) and water as negative control (NC). (a) The absorbance of the ABTS-polymer solutions over the first 3 h. The initial absorbance values shown correspond to the first measurable time point rather than *t* = 0 min due to a handling delay (∼1 min) between ABTS addition and UV–vis measurement. The observed differences reflect variations in radical scavenging rates among the samples. (b) Photographs of the cuvettes containing the ABTS-polymer solutions after 24 h incubation and (c) comparison of the relative ABTS radical scavenging by the tested polymers after 30 min and 24 h of incubation.

The ability of the copolymers to scavenge radicals is attributed to the thioether groups incorporated into the polyglycerol scaffold. However, GOTO and GTA exhibit different structural environments around the thioether functionality (Scheme 1). In GTA, the thioether is positioned within a more electron-deficient environment due to the proximity of two ester groups, which may influence the reactivity and accessibility of the sulfur center. In contrast, GOTO contains only one ester group per comonomer unit with greater spatial separation from the thioether moiety, resulting in a different electronic environment. These distinct structural arrangements presumably contribute to the differences in radical scavenging efficiency between GTA and GOTO (Figure 5a), with GTA demonstrating greater antioxidant activity.

The observed radical scavenging activity of the copolymers is promising for applications where oxidative stress plays a major role, such as in inflammatory conditions.^33,37^ However, the ABTS assay uses a synthetic radical and therefore only provides indicative value for the complex ROS/RNS interactions that occur in vivo.

### 3.6 Inhibition of Complement Activation

Multiple studies demonstrated that dPGS exhibits potent antiinflammatory properties and effectively inhibits complement system activation.^29,39,40^ Based on this, we evaluated the inhibitory potential of our newly developed copolymer platforms on the mannose-binding lectin (MBL) pathway of the complement activation. Therefore, an ELISA-based assay was applied. Mannan-coated plates were used to trigger the complement cascade in human serum. The assay quantifies complement activation by detecting membrane attack complexes (MAC), whereby lower levels of MAC indicate inhibition of the complement cascade. Heparin was included as a reference control for complement inhibition.^49^

As expected, all nonsulfated polymers, GOTO, GTA, and dPG, demonstrated minimal to no inhibition of the MBL pathway (Figure 6). In contrast, all sulfated derivatives showed a concentration-dependent inhibition, GTA-S was the most potent inhibitor with an IC_50_ value of 107 nM and 11% residual complement activity at 1000 nM. GOTO-S exhibited slightly lower potency with an IC_50_ of 155 nM and 24% residual activity. Its inhibitory profile was comparable to that of the reference controls, dPGS and heparin, which showed IC_50_ values of 174 nM and 151 nM, and residual activities of 19% and 22%, respectively.

**Figure 6:**
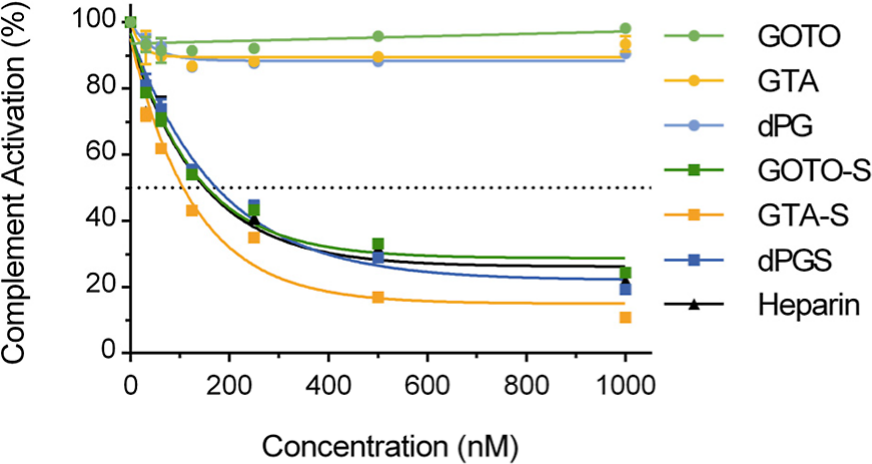
Concentration-dependent inhibition of the complement activation via the MBL pathway by the sulfated polymers (GOTO-S, GTA-S, dPGS) and heparin as reference control in contrast to the nonsulfated polymers (GOTO, GTA, dPG). Mean ± SEM, *n* = 2 (technical triplicates).

These results are consistent with prior studies on dPG and dPGS which highlight the importance of anionic sulfate groups for the inhibition of complement activation.^40^ The effective inhibition by GTA-S and GOTO-S suggests that they exhibit further sulfate-mediated therapeutic activities known for dPGS, supporting their potential as antiinflammatory polymer therapeutics.

## 4 Conclusion

Two distinct hyperbranched copolymers were synthesized from glycidol and a comonomer, OTO or TA, thereby introducing thioether as well as ester groups to the polymeric backbone. Characterization of the copolymers, GOTO and GTA, confirmed 10 mol % comonomer incorporation, molecular weights of 10 kDa, a hyperbranched architecture, and demonstrated the colloidal stability of the slightly negatively charged unimolecular particles in an aqueous environment. Sulfation of the copolymers yielded GOTO-S and GTA-S with a degree of sulfation around 80% and a stronger negative surface charge in aqueous solution. Cell viability and hemolysis assays indicated good in vitro biocompatibility, as all four derivatives were well tolerated by cells at concentrations up to 1 mg mL^–1^. The degradation through hydrolytic cleavage of ester groups was assessed over 4 weeks. GTA demonstrated faster degradation than GOTO, which is due to the double number of ester groups in TA compared to OTO units. Moreover, the structural differences of the comonomer units also influenced their ABTS scavenging efficiency. While all four copolymer derivatives exhibited radical scavenging activity in contrast to thioether-free controls, dPG and dPGS. Polymers containing TA showed faster radical scavenging, outperforming OTO-containing copolymers. The introduced sulfate groups in GOTO-S as well as GTA-S inhibited complement activation within a similar concentration range as the reference compounds dPGS and heparin. Among the polymers tested, GTA-S stood out due to its strong radical scavenging activity combined with a pronounced effect on complement activation. Both are beneficial properties for applications addressing oxidative stress in the context of inflammation. These findings provide insights into possible design strategies for polyglycerol platforms with intrinsic activity, stimuli-responsiveness, as well as targeting groups selected for a specific field of indication.

## Supplementary Material



## Notes

Additional characterization data and experimental details, including equations for calculation of degree of sulfation (dS), degree of branching (DB), ester cleavage (degradation); MS and ^1^H NMR spectra of the synthesized OTO monomer as well as ^1^H, ^13^C and 2D NMR spectra of the synthesized copolymers GOTO and GTA (PDF)

## §

Department Safety of Structures, Federal Institute for Materials Research and Testing, 12205 Berlin, Germany
