# Supplementary material for: Biodegradable Polyglycerols Combining Antioxidant Activity and Sulfation-Induced Complement Inhibition
Source: Biomacromolecules. 2025 Dec 11;27(1):439–50. doi: 10.1021/acs.biomac.5c01615 (PMC12801186; doi:10.1021/acs.biomac.5c01615)
Supplement: Supplementary file 1 [file bm5c01615_si_001.pdf]

Supporting Information

Biodegradable Polyglycerols Combining Antioxidant Activity and Sulfation-Induced Complement Inhibition

Hanna Koeppe[‡,a], *Daniel Horn*[‡,a], *Jens Dernedde*[b], *Rainer Haag**[a]

[a] Institute of Chemistry and Biochemistry, Freie Universität Berlin, 14195 Berlin, Germany

[b] Institute for Laboratory Medicine, Clinical Chemistry and Pathobiochemistry, Charité-Universitätsmedizin Berlin, 13353 Berlin, Germany

‡These authors contributed equally.

*Address correspondence to

Prof. Dr. Rainer Haag

Institute of Chemistry and Biochemistry

Freie Universität Berlin

14195 Berlin

Germany

E-Mail: haag@chemie.fu-berlin.de.

Table of Contents

SYNTHESIS OF 1,4-OXATHIEPAN-7-ONE (OTO) 3

Scheme S1. 3

SULFATION OF GOTO AND GTA 4

Scheme S2. 4

Equation S1 5

SUPPLEMENTAL CHARACTERIZATION DATA 6

Figure S2. 6

Figure S3. 7

Equation S2. 7

Figure S4. 8

Figure S5. 8

Figure S6. 9

Equation S3. 9

Figure S8. 10

SUPPLEMENTAL MS AND NMR SPECTRA 11

REFERENCES 19

SYNTHESIS OF 1,4-OXATHIEPAN-7-ONE (OTO)

Scheme S1. Synthesis of 1,4-oxathiepan-7-one (OTO): (a) condensation reaction to form *p*-nitrophenol acrylate followed by (b) an one-pot two-step reaction including a thia-Michael addition and an intramolecular cyclization. Synthesis adapted from Li *et al.*[1]

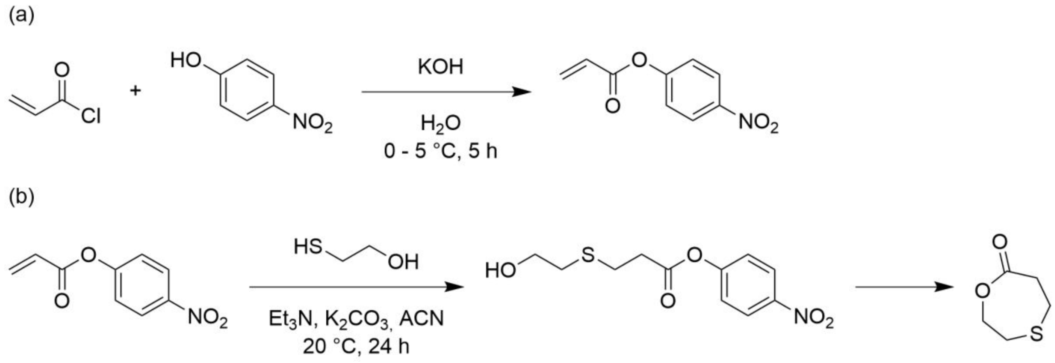

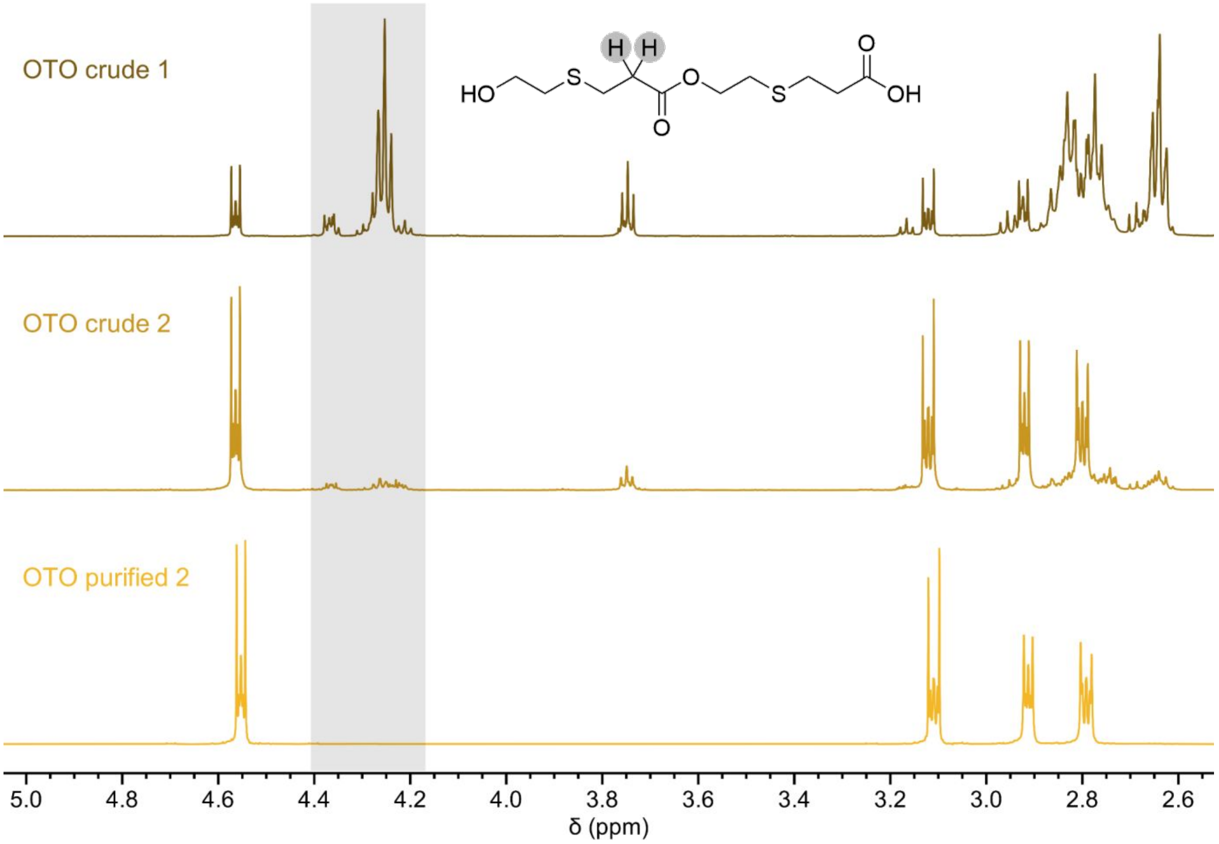

Figure S1. ^{1}H-NMR spectra ($CDCl_3$, 500 MHz) of OTO synthesized *via* literature procedure[1] (top) or the optimized procedure with pseudo-dilution at room temperature before (middle), and after purification (bottom). Signals of oligomerization products are highlighted.

SULFATION OF GOTO AND GTA

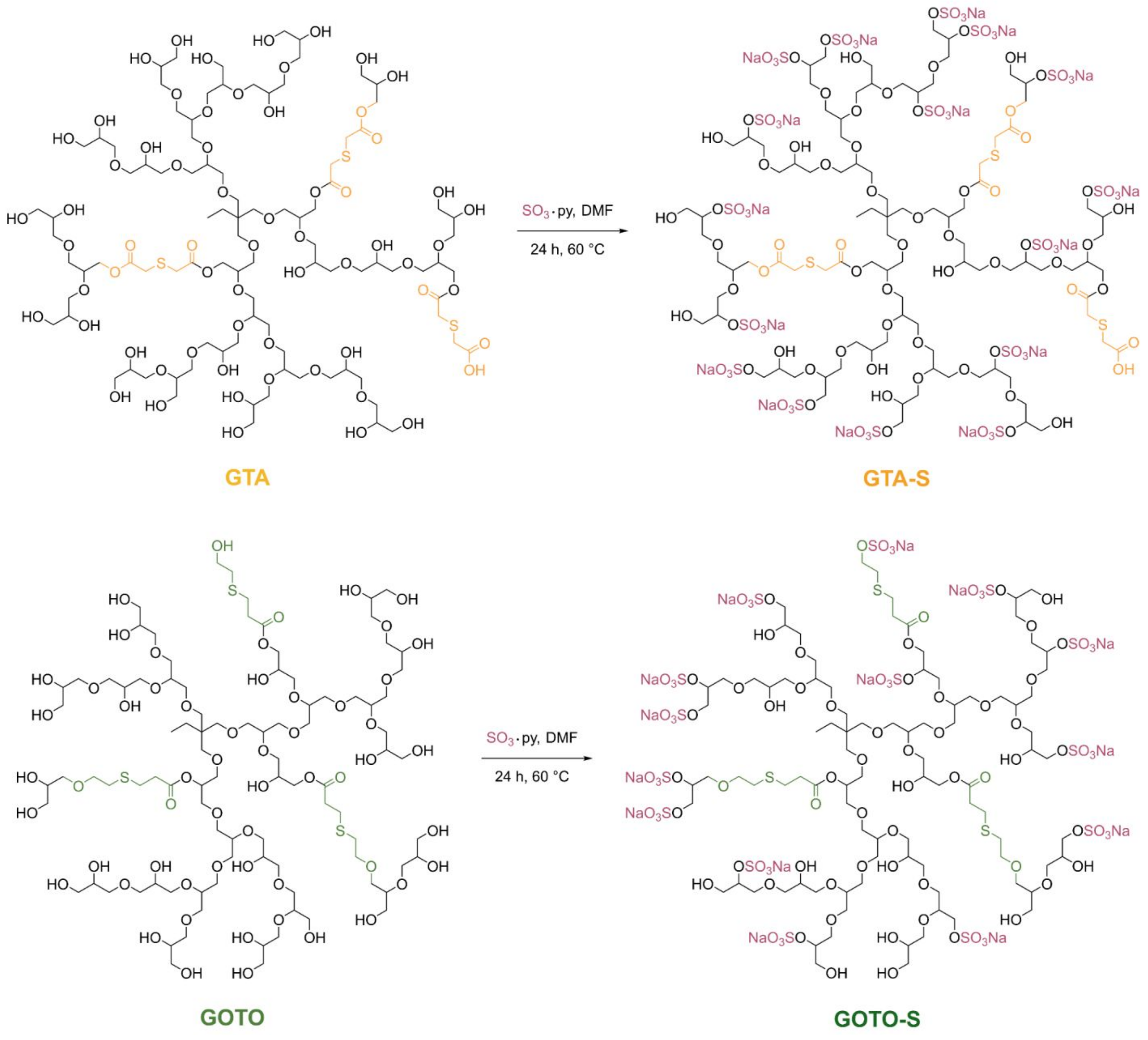

Scheme S2. Sulfation of GTA and GOTO to form GTA-S and GOTO-S. Structures are shown as representative examples.

Equation S1. Calculation of the degree of sulfation (dS) based on the sulfur content measured by EA exemplified for GTA-S.

$$\boldsymbol{dS = \frac{\%S - \%S_{GTA}}{\%S_{GTA-S_{100\%}} - \%S_{GTA}}}$$

$$\boldsymbol{dS = \frac{\%S - \frac{\%TA \times MW_S}{\%G \times MW_G + \%TA \times MW_{TA}} \times 100}{\frac{MW_S}{\%G \times \left(MW_G + MW_{SO_3Na} - MW_H\right) + \%TA \times MW_{TA}} - \frac{\%TA \times MW_S}{\%G \times MW_G + \%TA \times MW_{TA}} \times 100}}$$

$\%\boldsymbol{S}$: mean sulfur content measured by EA; $\%\boldsymbol{S_{GTA}}$: sulfur content in GTA; $\%\boldsymbol{S_{GTA-S_{100\%}}}$: calculated sulfur content in GTA-S with a dS of 100%; $\%\boldsymbol{TA}$: comonomer (TA) content in GTA; $\%\boldsymbol{G}$: glycidol content in GTA; $\boldsymbol{MW}$: molecular weight of the subscripted molecule or atom.

SUPPLEMENTAL CHARACTERIZATION DATA

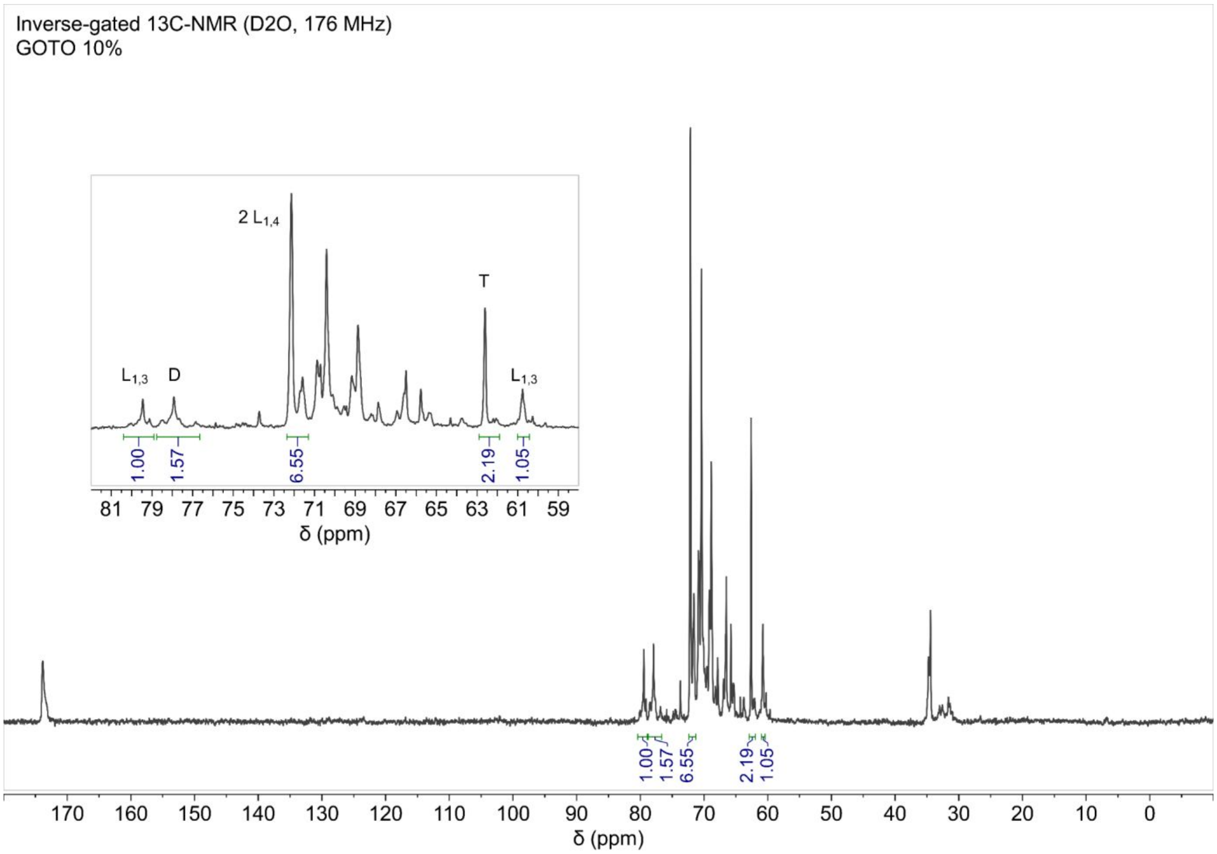

Figure S2. Inverse-gated ^{13}C-NMR (D_2O, 172 MHz) spectrum of GOTO. The introduced comonomer led to increased spectral complexity compared to dPG, which prevented unambiguous assignment of all signals according to the nomenclature described by Sunder *et al.*[2] Signals confidently attributed to terminal (T), dendritic (D), linear 1,3 ($L_{1,3}$), and linear 1,4 ($L_{1,4}$) units were labeled accordingly and used for DB calculation (Equation S2).

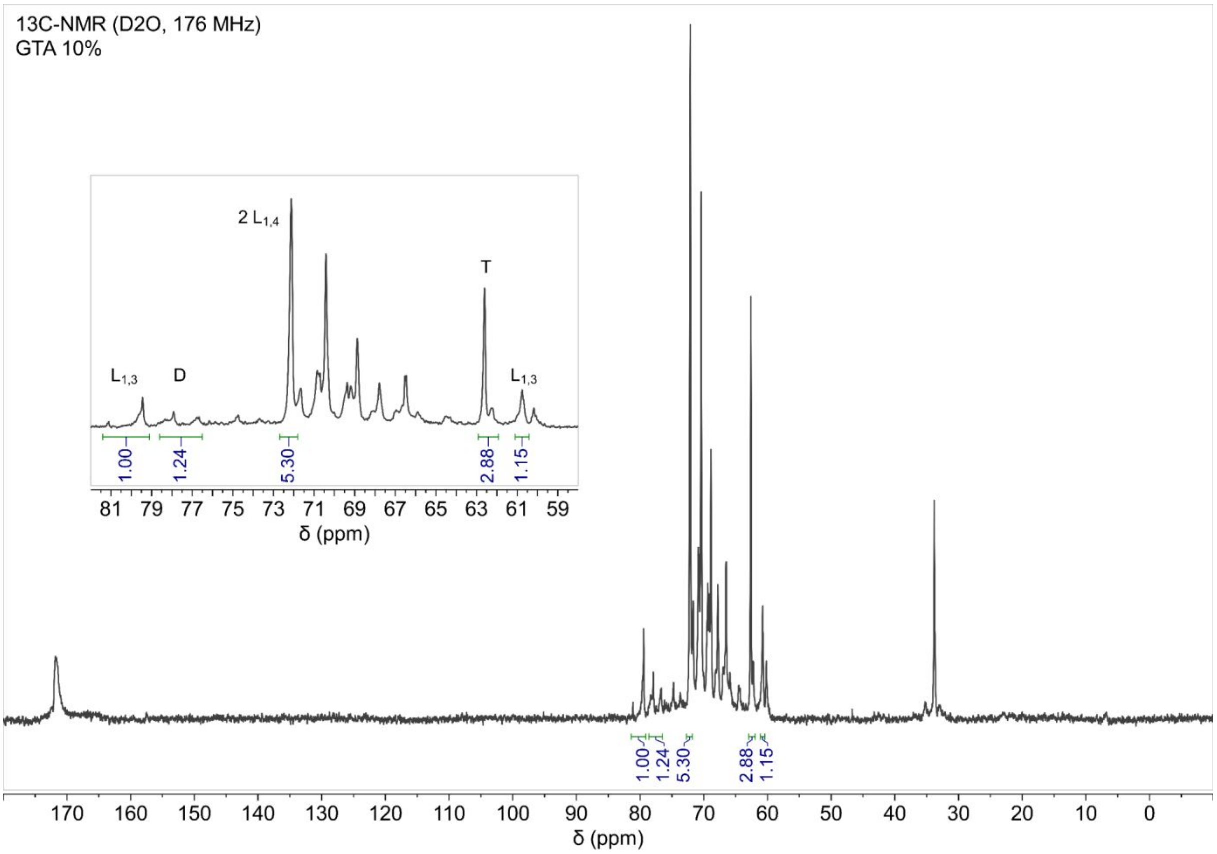

Figure S3. Inverse-gated ^{13}C-NMR (D_2O, 172 MHz) spectrum of GTA. The introduced comonomer led to increased spectral complexity compared to dPG, which prevented unambiguous assignment of all signals according to the nomenclature described by Sunder *et al.*[2] Signals confidently attributed to terminal (T), dendritic (D), linear 1,3 ($L_{1,3}$), and linear 1,4 ($L_{1,4}$) units were labeled accordingly and used for DB calculation (Equation S2).

Equation S2. Calculation of the degree of branching (DB)[2,3] based on the respective integrals in the iG ^{13}C-NMR of GTA and GOTO (Figure S2-S3). D = dendritic; $L_{1,3}$ = linear 1,3; $L_{1,4}$ = linear 1,4.

$$DB = \frac{2D}{2D + L_{1,3} + L_{1,4}}$$

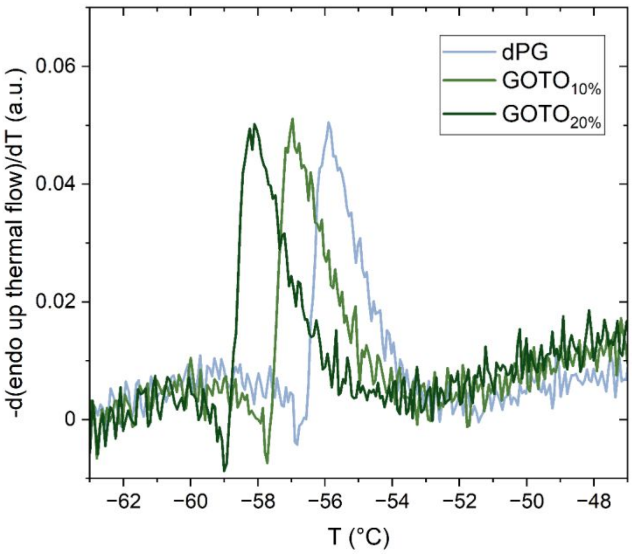

Figure S4. Differential scanning calorimetry (DSC) of GOTO with 10% and 20% OTO content in comparison to dPG. Data obtained from the 2nd heating cycle.

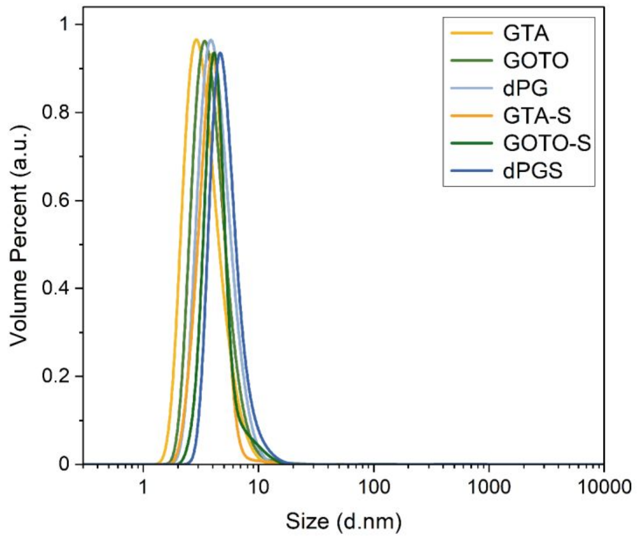

Figure S5. DLS graphs of the non-sulfated and sulfated copolymers in comparison to dPG and dPGS in PBS (pH 7.4) at 2 mg mL^{-1}.

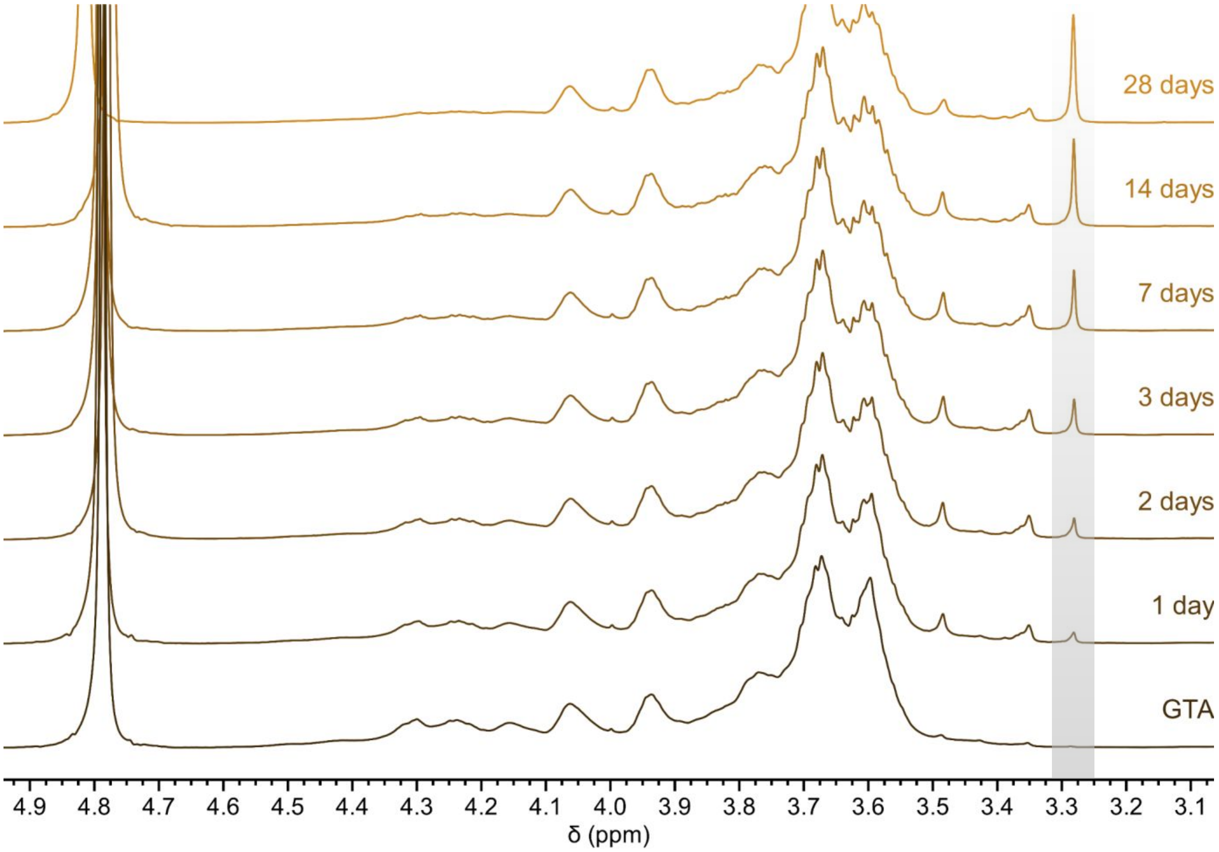

Figure S6. Degradation of GTA in PBS (pH 7.4) at 37 ˚C observed by ^{1}H-NMR spectroscopy (500 MHz, D_2O) after different time periods. Representative data from one of two independent experiments.

Equation S3. Calculation of the ester cleavage in GTA or GOTO based on the respective signals in their ^{1}H-NMR spectra after different time periods.

$$\boldsymbol{ester\ cleavage}\ (\%) = \frac{\boldsymbol{I_{t_x}} - \boldsymbol{I_{t_0}}}{\boldsymbol{I_{100\%}} - \boldsymbol{I_{t_0}}}$$

$\boldsymbol{I_{t_x}}$: Integral after x days; $\boldsymbol{I_{t_0}}$: integral after 0 d; $\boldsymbol{I_{100\%}}$; calculated integral for 100% ester cleavage.

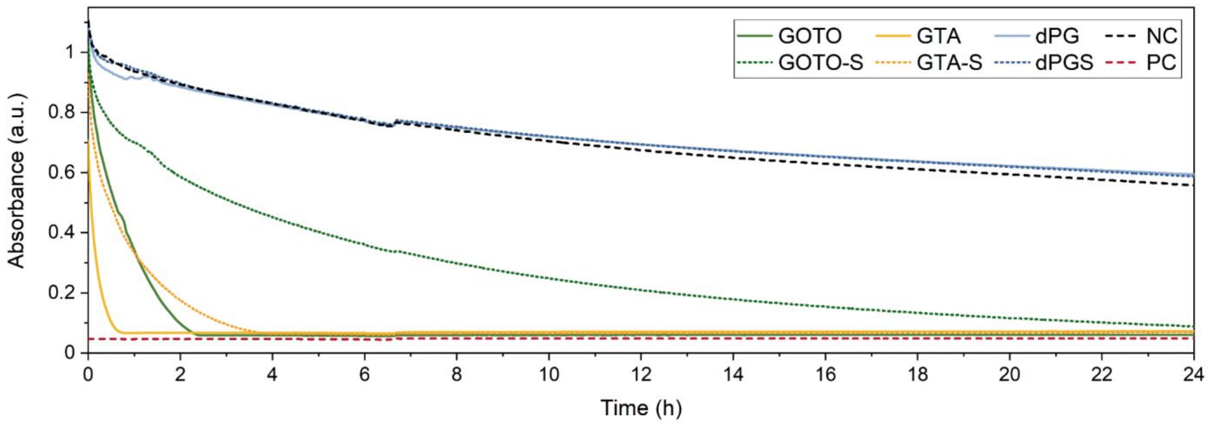

Figure S7. ABTS radical scavenging[4] by the copolymers as well as dPG and dPGS, quantified by UV/Vis absorption spectroscopy over 24 h. Vitamin C (50 µM) served as a positive control (PC) and water as negative control (NC).

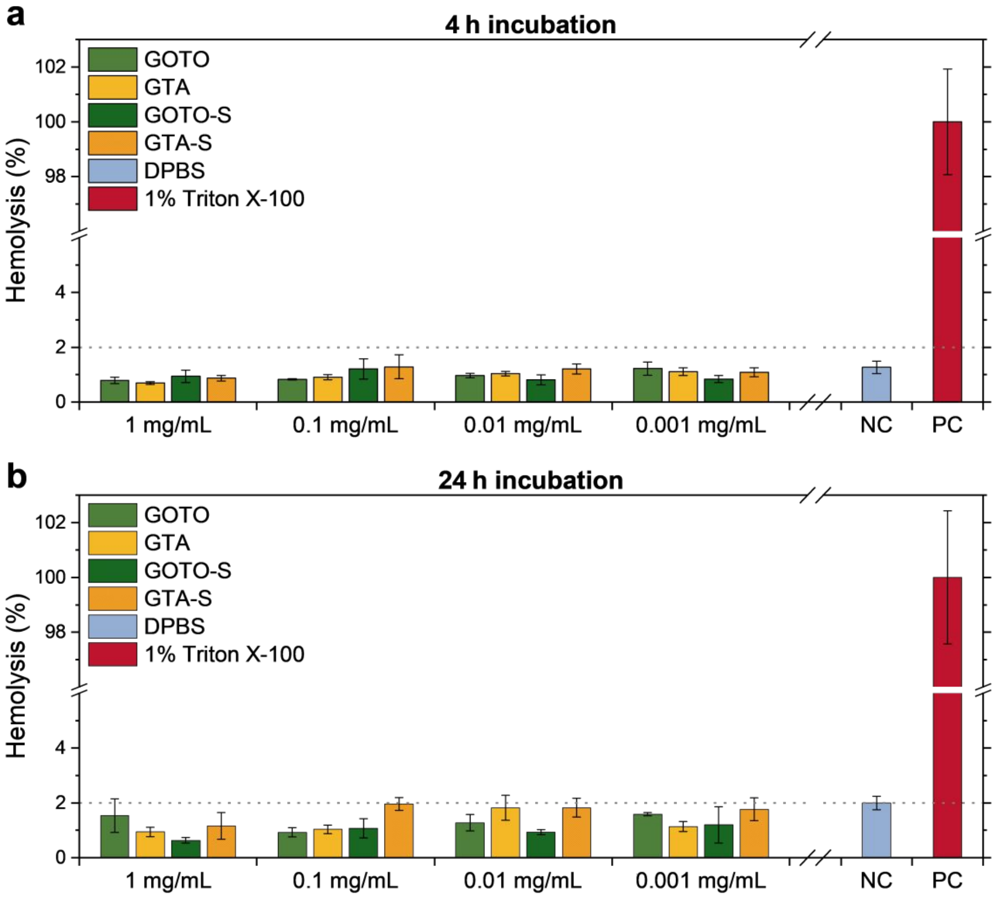

Figure S8. Results of the *ex vivo* red blood cell (RBC) hemolysis assay[5] after 4 h (a) and 24 h (b) incubation with different concentrations of the copolymers. Data are shown in comparison to

DPBS (negative control, NC) and 1% Triton X-100 (positive control, PC). Absorbance values were background-corrected using DPBS without RBCs and normalized to the PC.

Supplemental MS and NMR Spectra

HRMS of 1,4-oxathiepan-7-one (OTO)

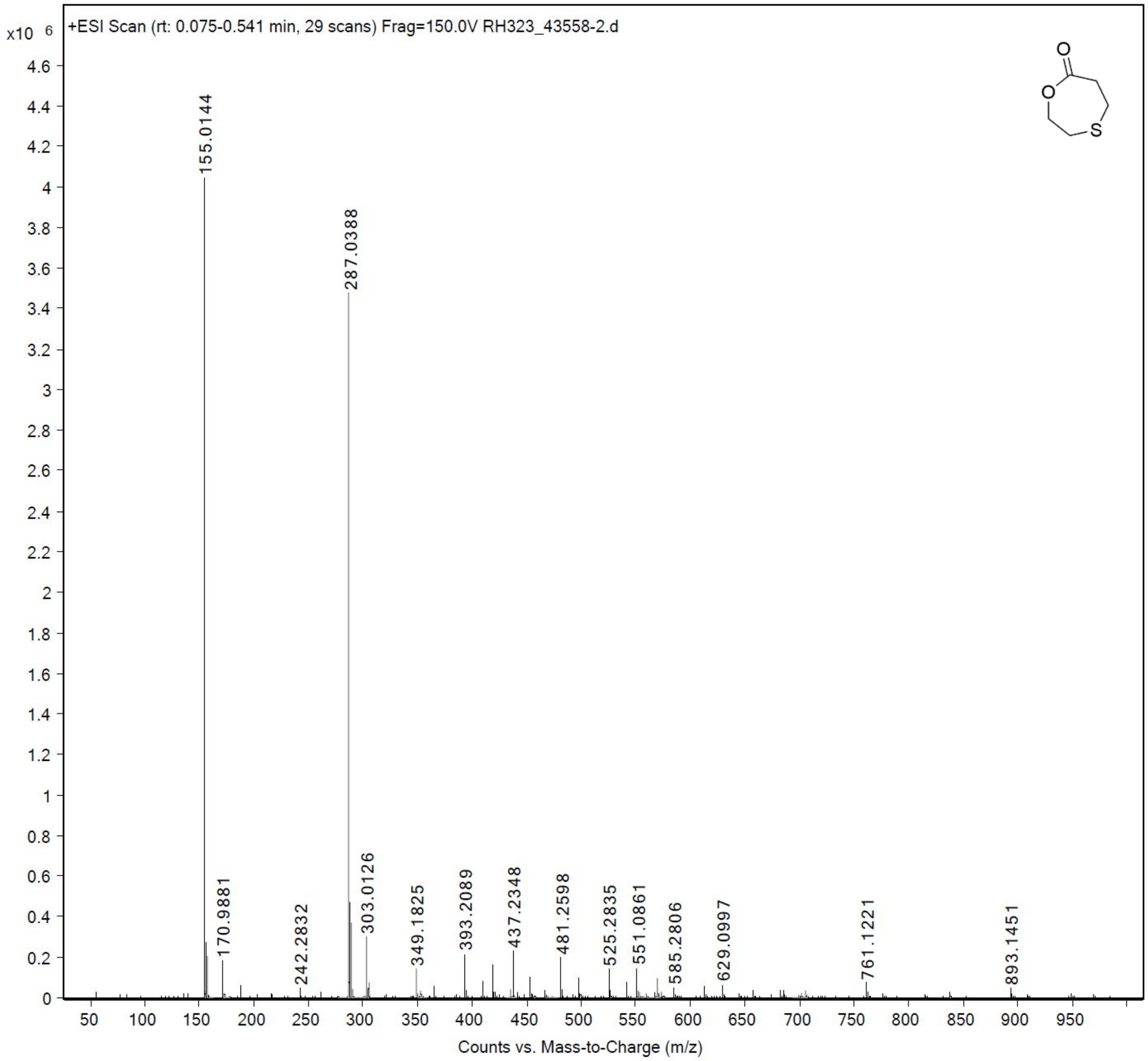

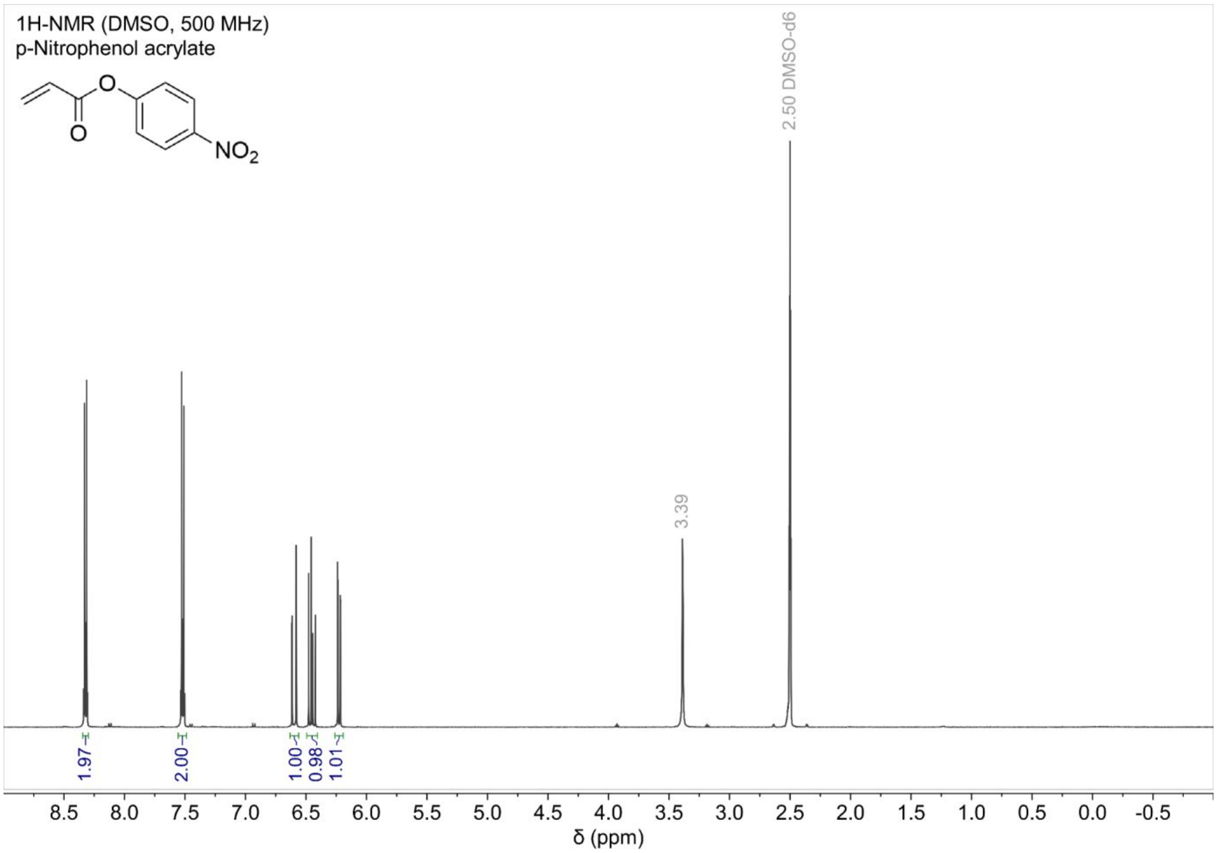
1H-NMR (DMSO, 500 MHz)
p-Nitrophenol acrylate
NO_2
2.50 DMSO-d6
3.39
1.97
2.00
1.00
0.98
1.01
8.5 8.0 7.5 7.0 6.5 6.0 5.5 5.0 4.5 4.0 3.5 3.0 2.5 2.0 1.5 1.0 0.5 0.0 -0.5
δ (ppm)

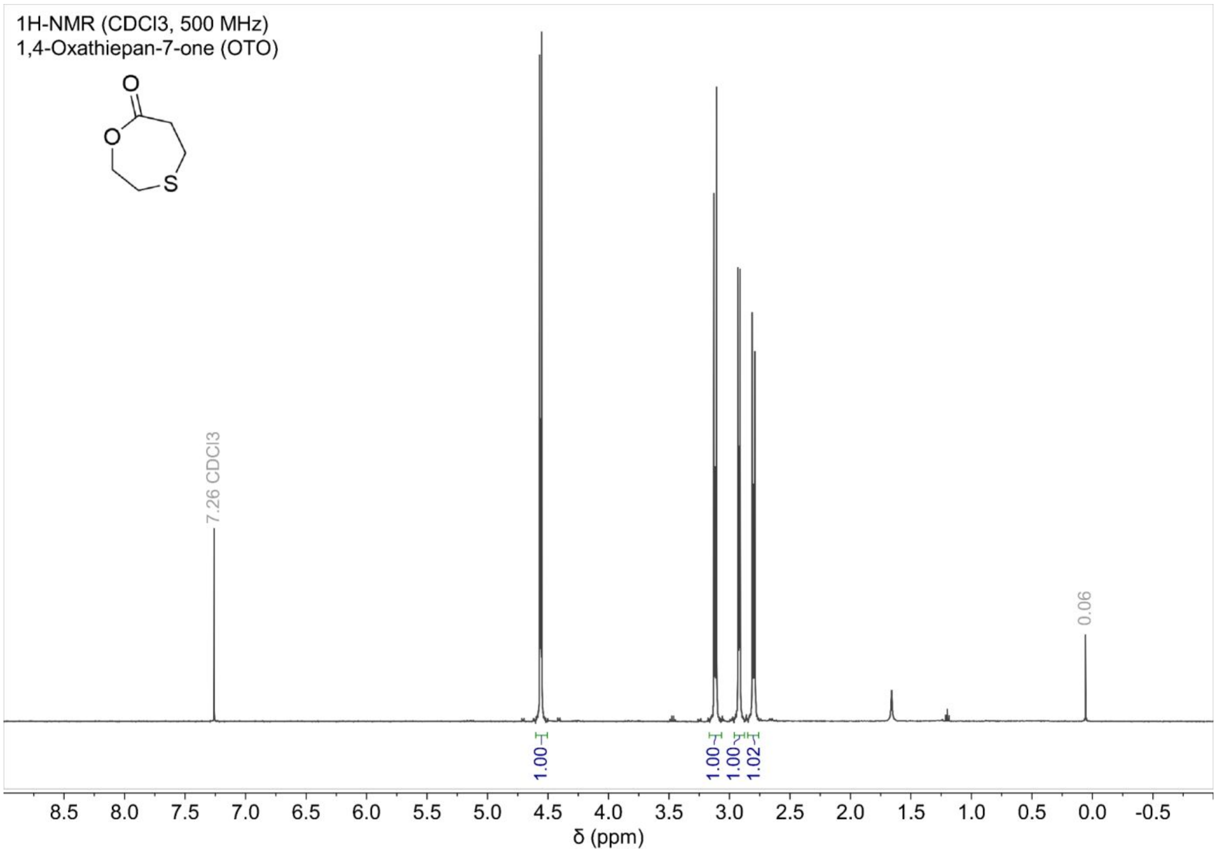
1H-NMR (CDCl3, 500 MHz)
1,4-Oxathiepan-7-one (OTO)
7.26 CDCl3
0.06
1.00
1.00
1.00
1.02
8.5 8.0 7.5 7.0 6.5 6.0 5.5 5.0 4.5 4.0 3.5 3.0 2.5 2.0 1.5 1.0 0.5 0.0 -0.5
δ (ppm)

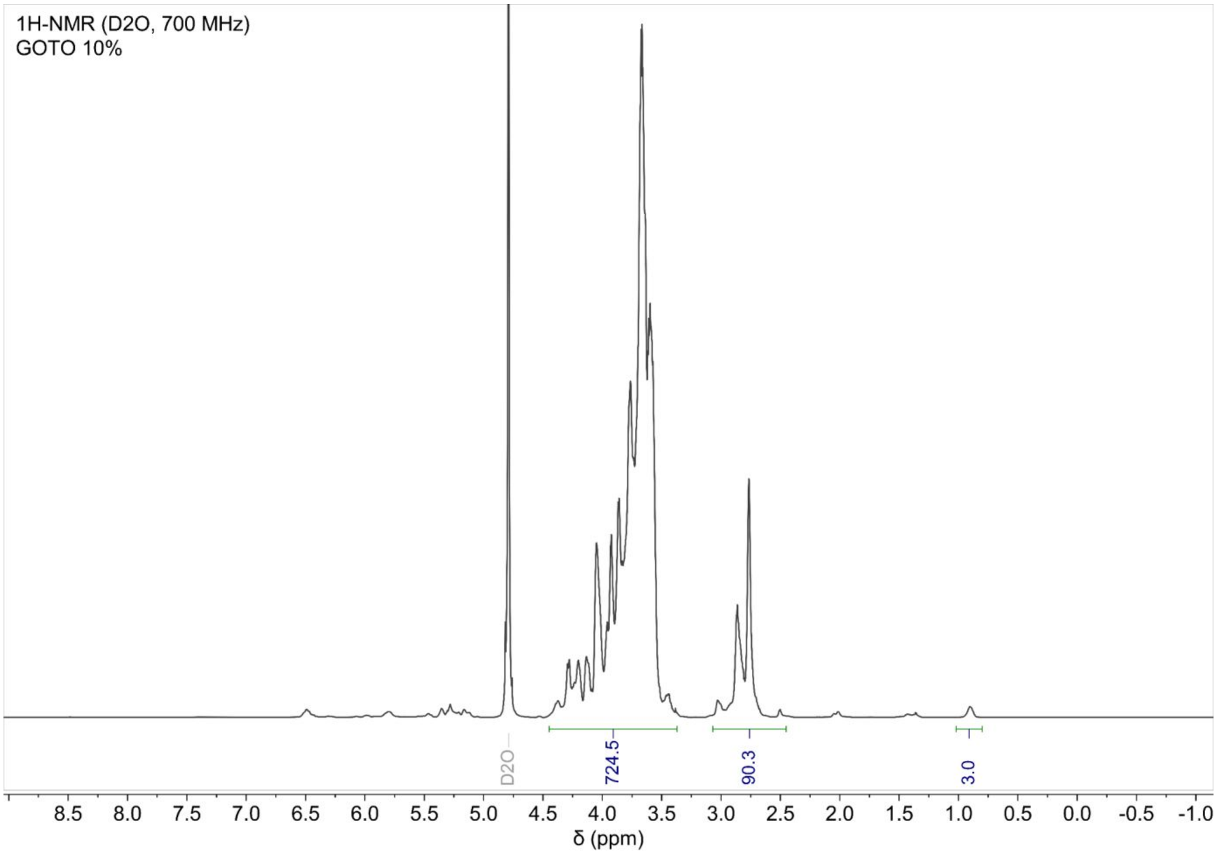
1H-NMR (D2O, 700 MHz)
GOTO 10%
D2O
724.5
90.3
3.0
8.5 8.0 7.5 7.0 6.5 6.0 5.5 5.0 4.5 4.0 3.5 3.0 2.5 2.0 1.5 1.0 0.5 0.0 -0.5 -1.0
δ (ppm)

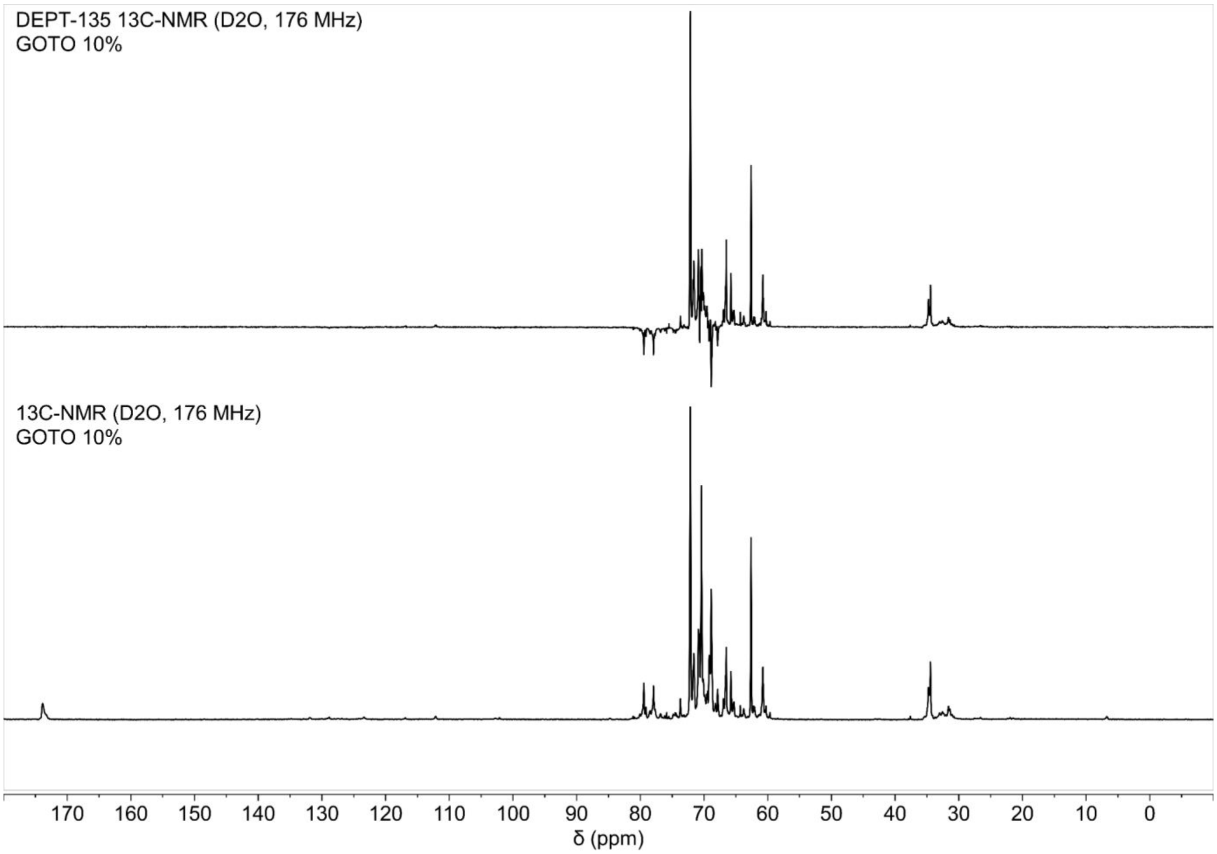
DEPT-135 13C-NMR (D2O, 176 MHz)
GOTO 10%
13C-NMR (D2O, 176 MHz)
GOTO 10%
170 160 150 140 130 120 110 100 90 80 70 60 50 40 30 20 10 0
δ (ppm)

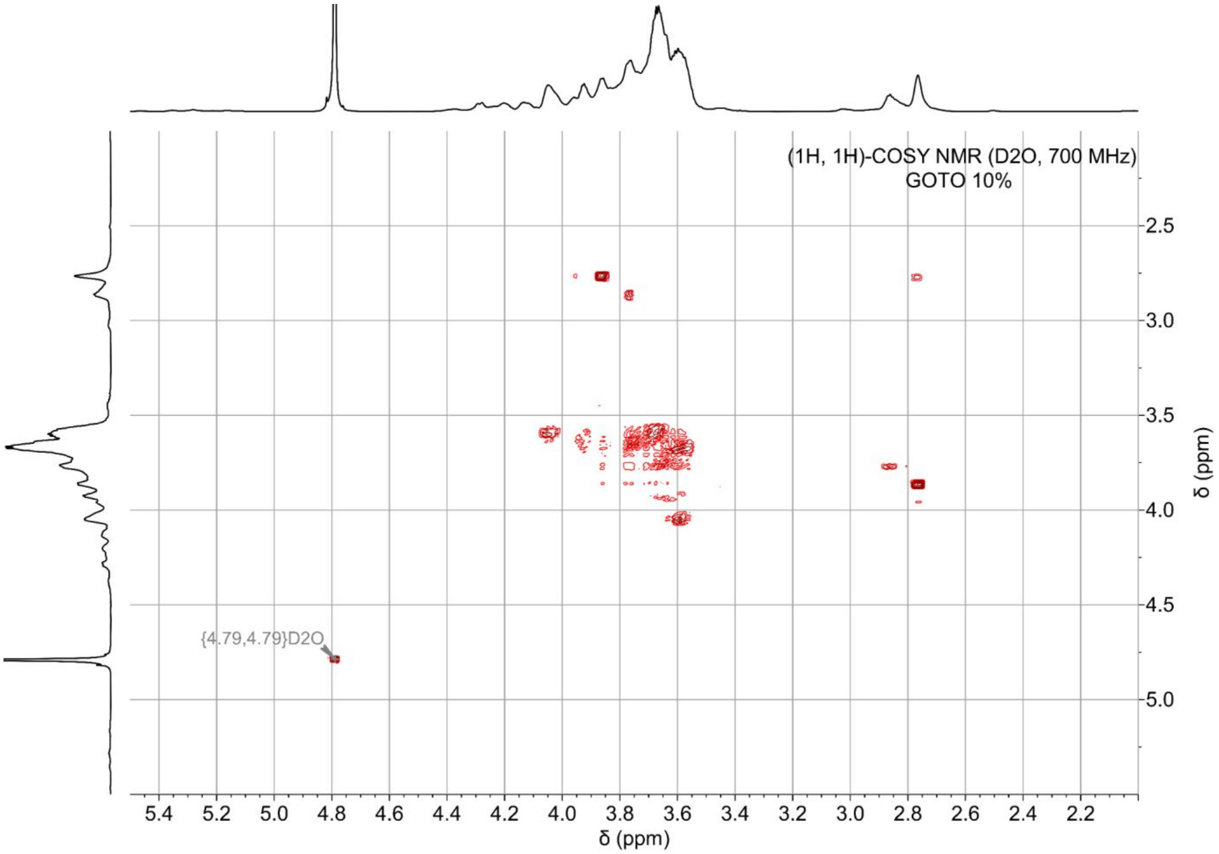
(1H, 1H)-COSY NMR (D2O, 700 MHz)
GOTO 10%
{4.79,4.79}D2O
δ (ppm)
δ (ppm)

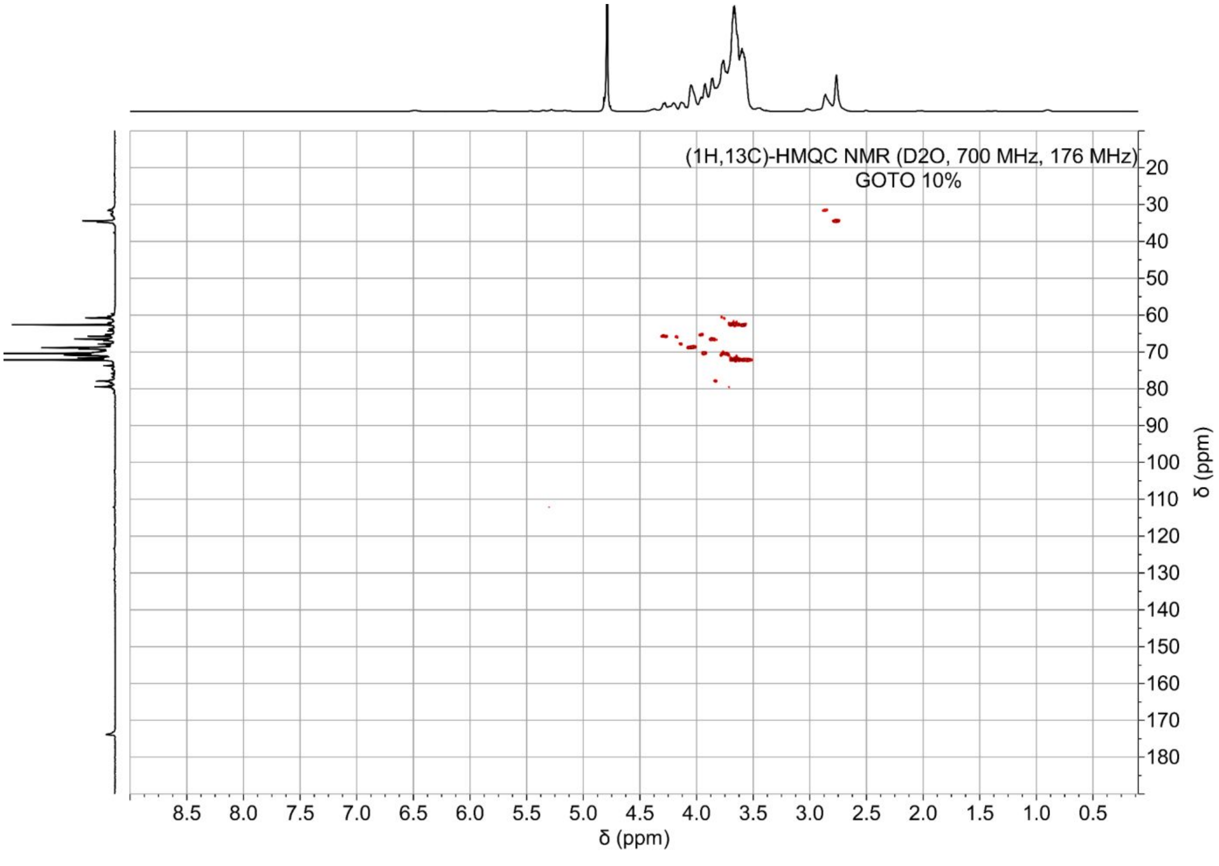
(1H,13C)-HMQC NMR (D2O, 700 MHz, 176 MHz)
GOTO 10%
δ (ppm)
δ (ppm)

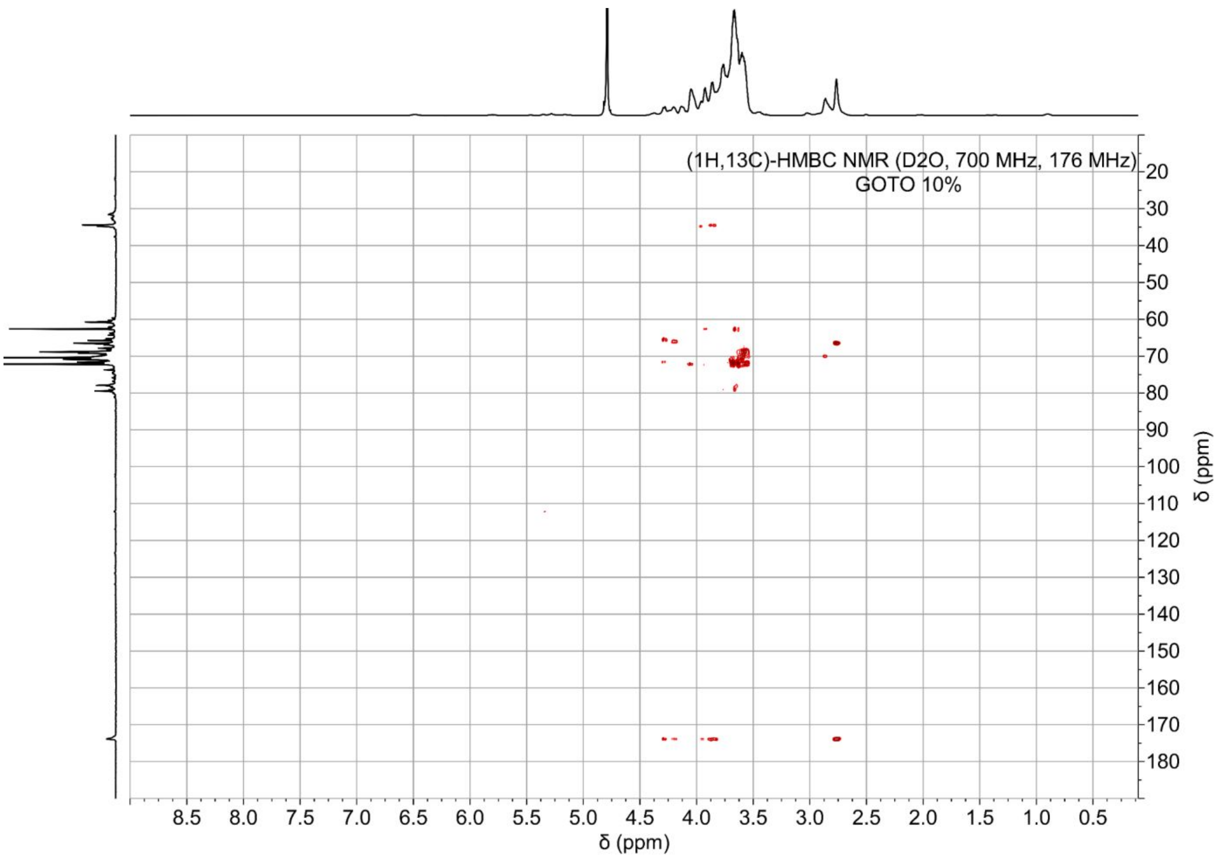
(1H,13C)-HMBC NMR (D2O, 700 MHz, 176 MHz)
GOTO 10%
δ (ppm)
δ (ppm)

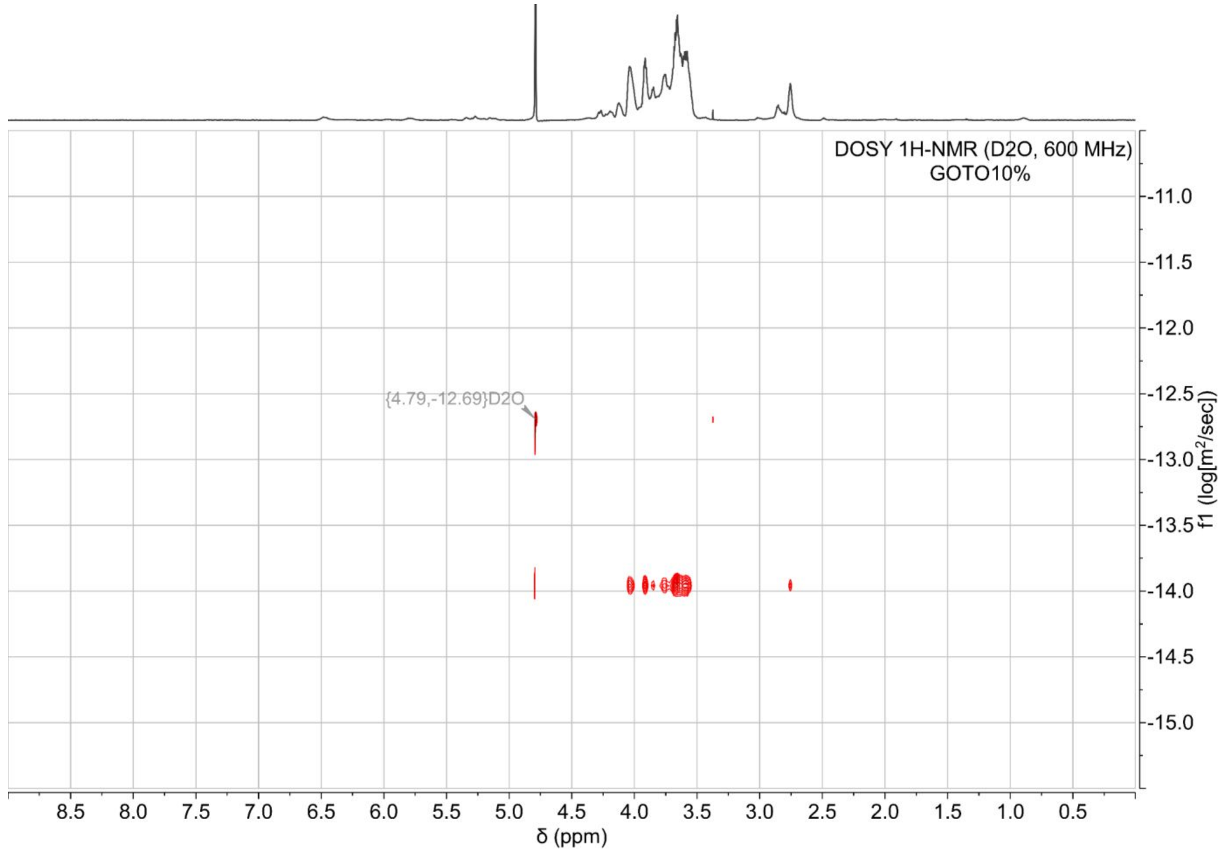
DOSY 1H-NMR (D2O, 600 MHz)
GOTO10%
{4.79,-12.69}D2O
f1 (log[m²/sec])
δ (ppm)

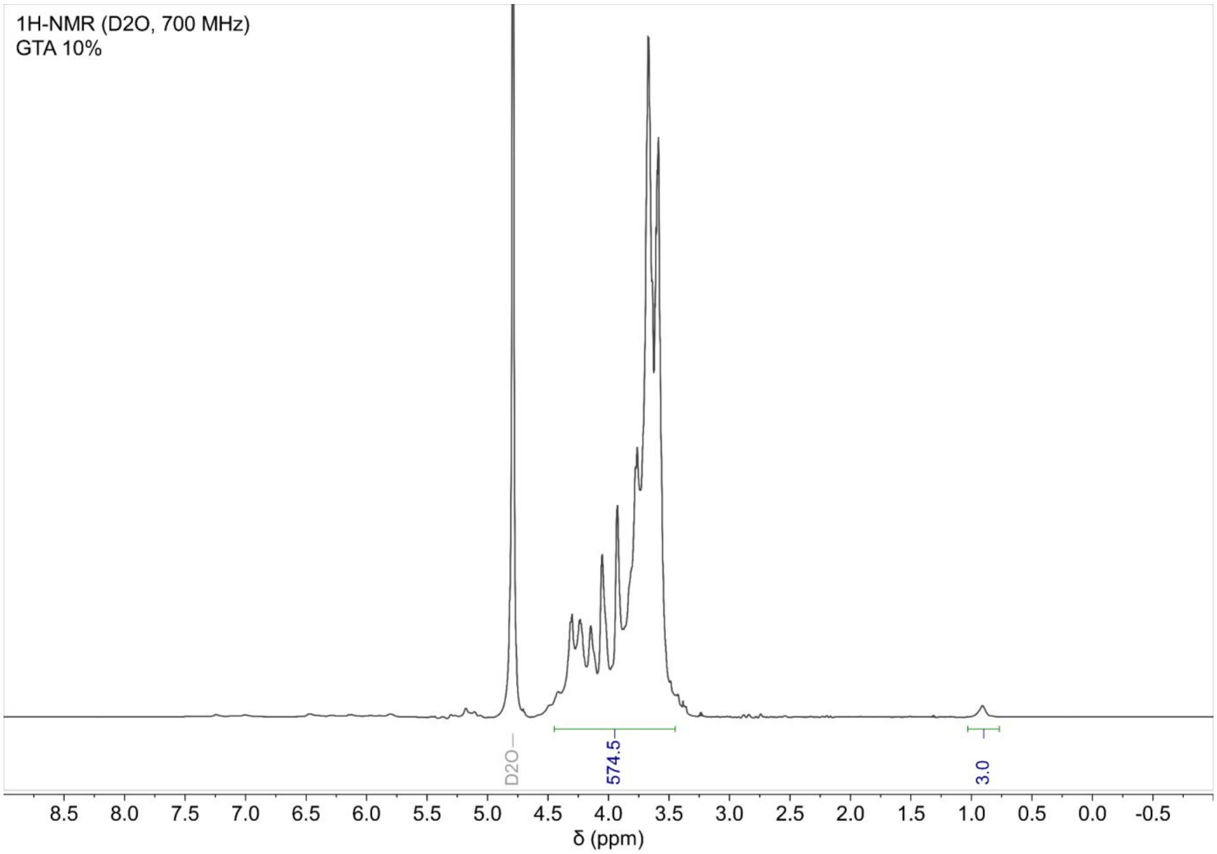

1H-NMR (D2O, 700 MHz)
GTA 10%
D2O
574.5
3.0
8.5 8.0 7.5 7.0 6.5 6.0 5.5 5.0 4.5 4.0 3.5 3.0 2.5 2.0 1.5 1.0 0.5 0.0 -0.5
δ (ppm)

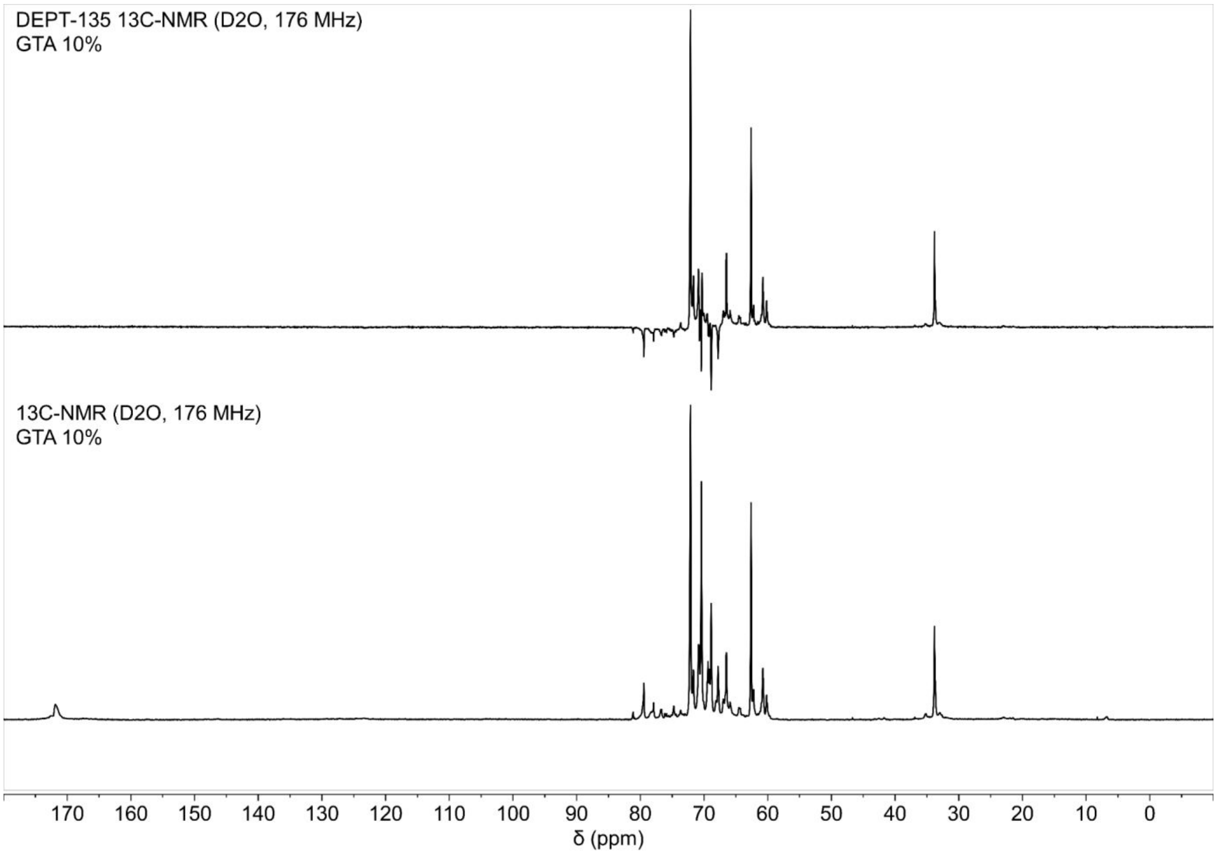

DEPT-135 13C-NMR (D2O, 176 MHz)
GTA 10%
13C-NMR (D2O, 176 MHz)
GTA 10%
170 160 150 140 130 120 110 100 90 80 70 60 50 40 30 20 10 0
δ (ppm)

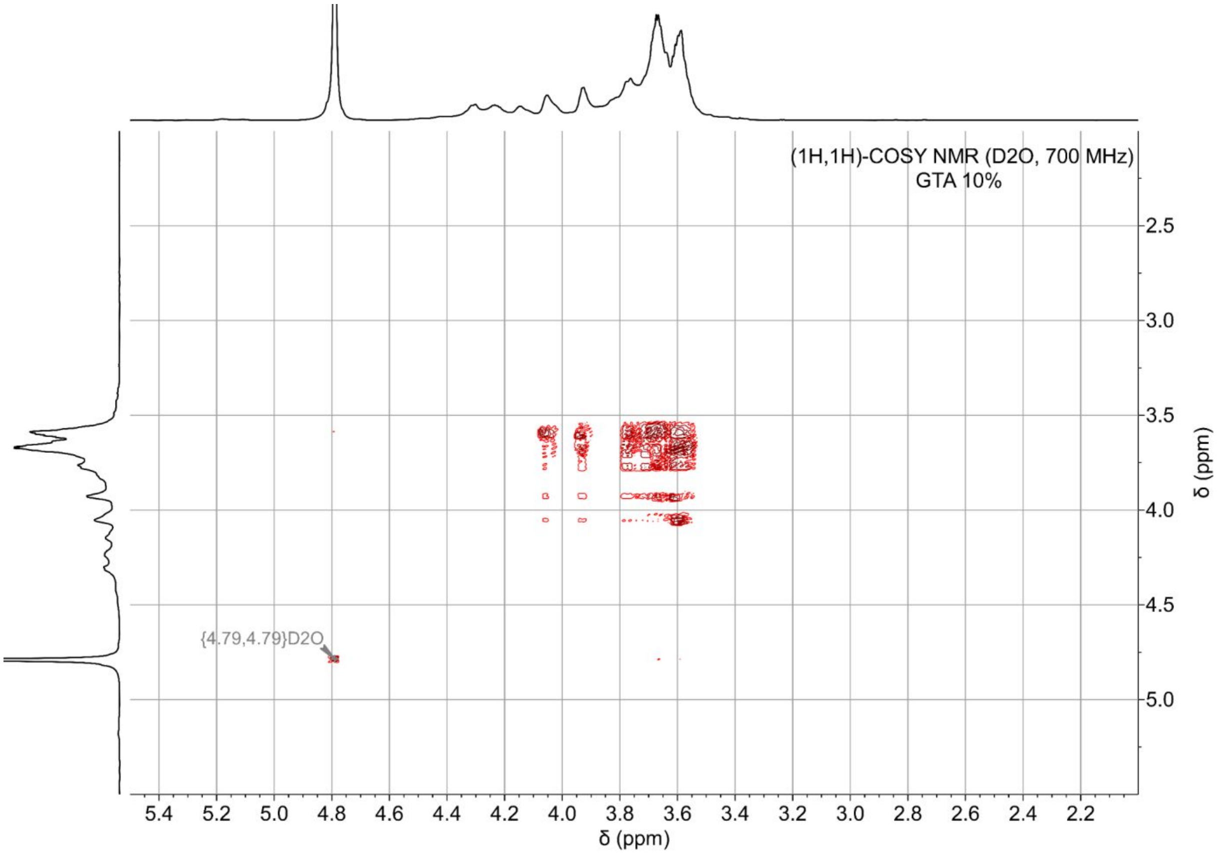
(1H,1H)-COSY NMR (D2O, 700 MHz)
GTA 10%
{4.79,4.79}D2O
δ (ppm)
δ (ppm)

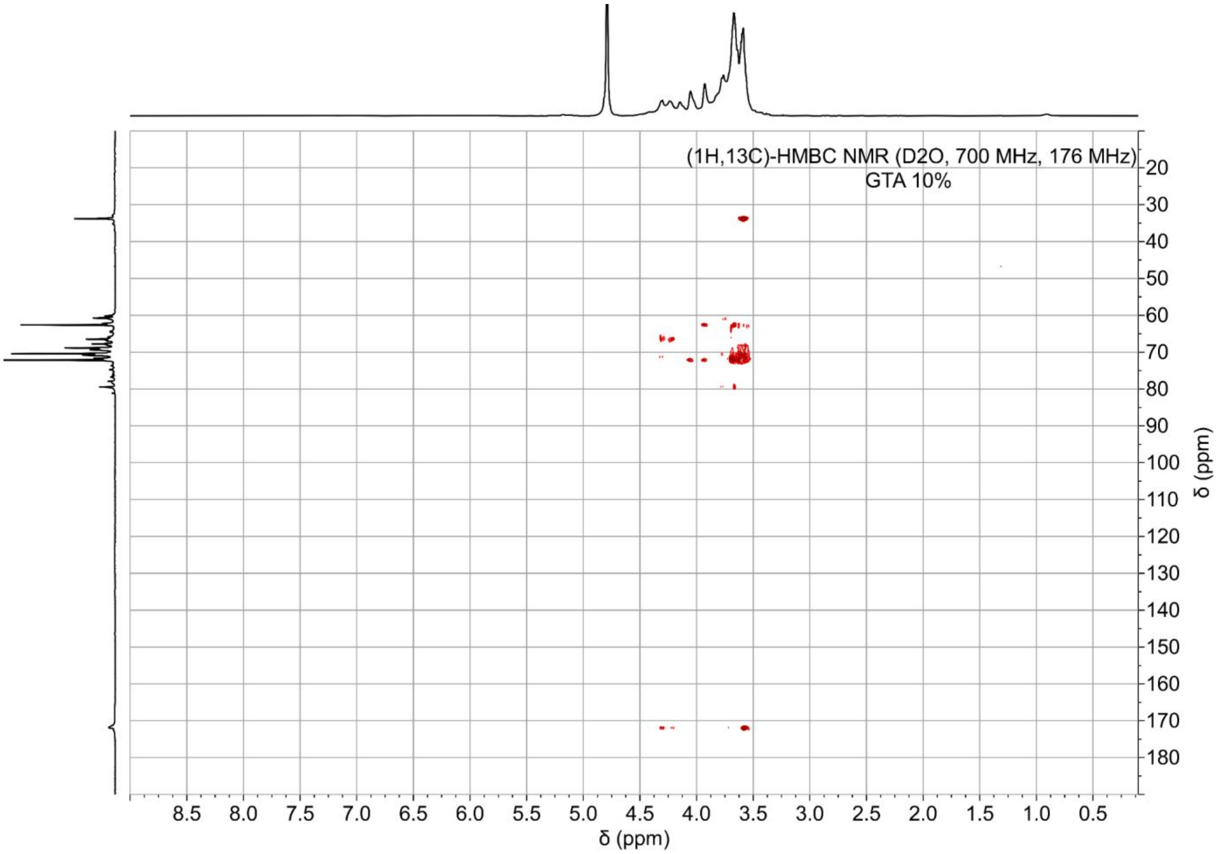
(1H,13C)-HMBC NMR (D2O, 700 MHz, 176 MHz)
GTA 10%
δ (ppm)
δ (ppm)

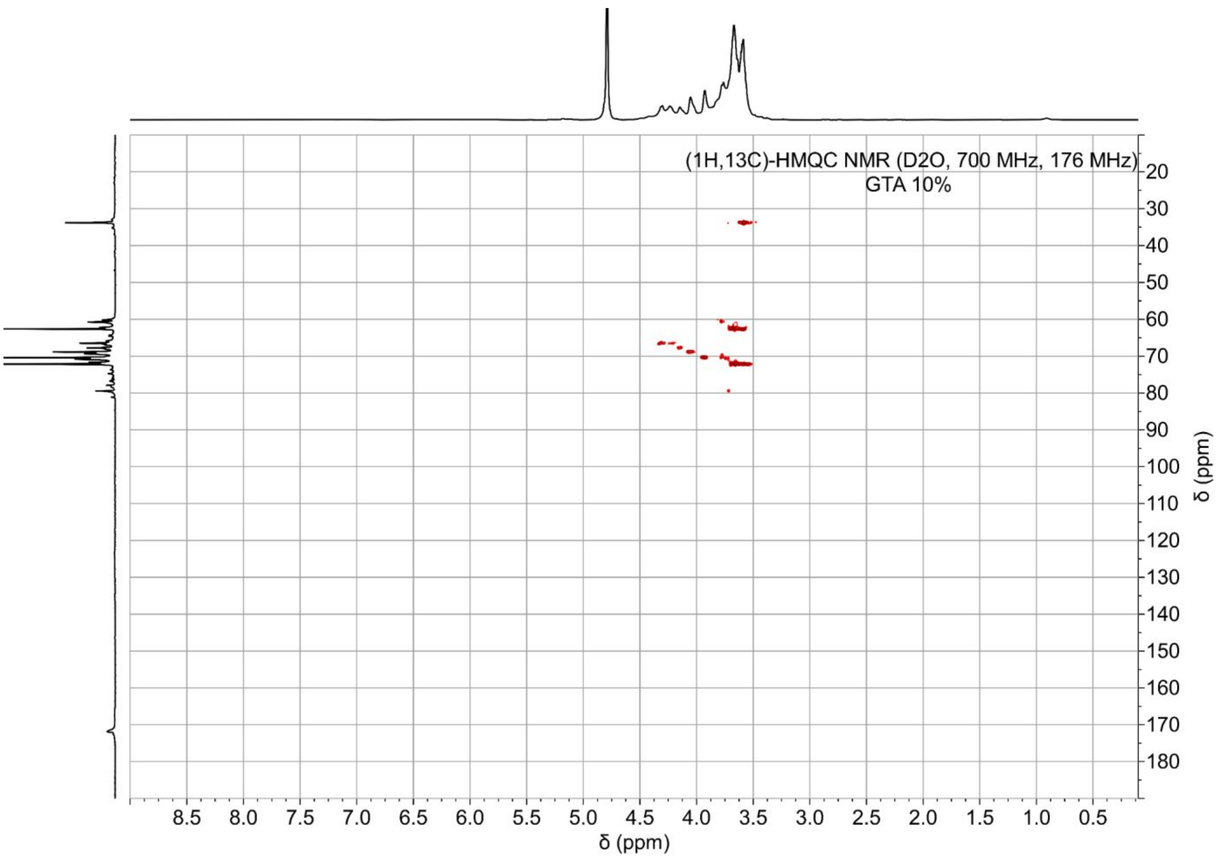
(1H,13C)-HMQC NMR (D2O, 700 MHz, 176 MHz)
GTA 10%
δ (ppm)
δ (ppm)

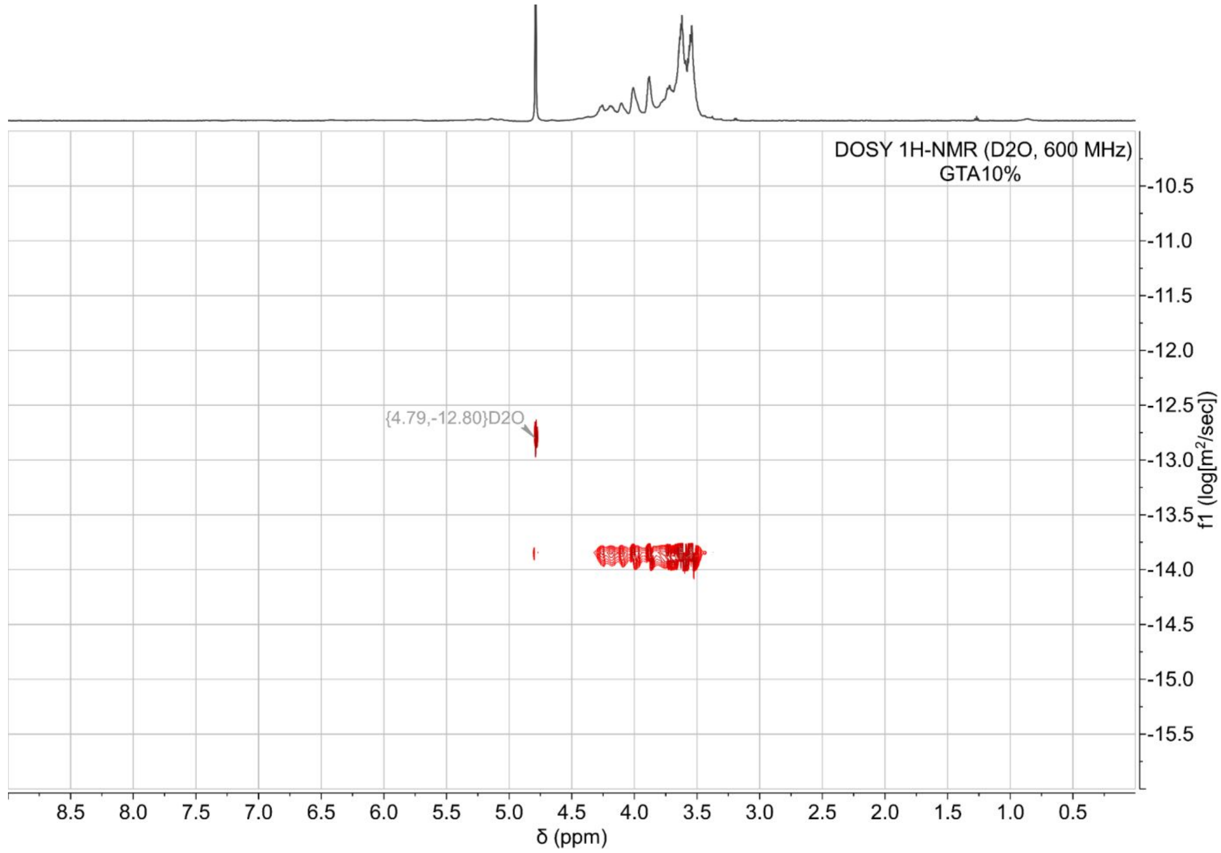
DOSY 1H-NMR (D2O, 600 MHz)
GTA10%
{4.79,-12.80}D2O
f1 (log[m²/sec])
δ (ppm)

REFERENCES

(1) Li, L.; Wang, Q.; Lyu, R.; Yu, L.; Su, S.; Du, F.-S.; Li, Z.-C. Synthesis of a ROS-responsive analogue of poly(ε-caprolactone) by the living ring-opening polymerization of 1,4-oxathiepan-7-one. *Polym. Chem.* **2018**, *9* (36), 4574–4584. DOI: 10.1039/C8PY00798E.

(2) Sunder, A.; Hanselmann, R.; Frey, H.; Mülhaupt, R. Controlled Synthesis of Hyperbranched Polyglycerols by Ring-Opening Multibranching Polymerization. *Macromolecules* **1999**, *32* (13), 4240–4246. DOI: 10.1021/ma990090w.

(3) Hölter, D.; Burgath, A.; Frey, H. Degree of branching in hyperbranched polymers. *Acta Polym.* **1997**, *48* (1-2), 30–35. DOI: 10.1002/actp.1997.010480105.

(4) Re, R.; Pellegrini, N.; Proteggente, A.; Pannala, A.; Yang, M.; Rice-Evans, C. Antioxidant activity applying an improved ABTS radical cation decolorization assay. *Free Radic. Biol. Med.* **1999**, *26* (9-10), 1231–1237. DOI: 10.1016/s0891-5849(98)00315-3.

(5) Evans, B. C.; Nelson, C. E.; Yu, S. S.; Beavers, K. R.; Kim, A. J.; Li, H.; Nelson, H. M.; Giorgio, T. D.; Duvall, C. L. Ex vivo red blood cell hemolysis assay for the evaluation of pH-responsive endosomolytic agents for cytosolic delivery of biomacromolecular drugs. *J. Vis. Exp.* **2013** (73), e50166. DOI: 10.3791/50166.
